# Supplementary material for: Systemically injected oxygen within rapidly dissolving microbubbles improves the outcomes of severe hypoxaemia in swine
Source: Nat Biomed Eng. 2024 Oct 17;8(11):1396–411. doi: 10.1038/s41551-024-01266-8 (PMC11584390; doi:10.1038/s41551-024-01266-8)
Supplement: Supplementary file 1 — Supplementary methods, tables, figures and video captions. [file 41551_2024_1266_MOESM1_ESM.pdf]

nature biomedical engineering

Supplementary information https://doi.org/10.1038/s41551-024-01266-8

Systemically injected oxygen within rapidly dissolving microbubbles improves the outcomes of severe hypoxaemia in swine

In the format provided by the authors and unedited

Contents

Materials and characterizations.

Supplementary table 1.

Supplementary figures 1–31.

Captions for Supplementary videos 1–5.

Materials and characterizations

Materials

Dextran (Mr 6,000, Leuconostoc spp.), 4-dimethyl aminopyridine (DMAP), acetic anhydride, succinic anhydride, dextrose (pharmaceutical grade), phosphate buffered saline, anhydrous dimethyl sulfoxide (DMSO), acetic acid were all purchased from Sigma and used as received. Zirconium-89 was purchased from Washington University – St. Louis. All water used was deionized water ultra-purified by a Milli-Q System (18 MΩ; Millipore). Medical grade oxygen and nitrogen were purchased from Airgas and used as received.

Physical Characterizations

^{1}H Nuclear magnetic resonance (^{1}H NMR) spectra were recorded in deuterated solvents on a 400 MHz magnet Varian spectrometer. Fourier transform infrared spectroscopy (FTIR) was conducted on a Spectrum One FT-IR Spectrometer (PerkinElmer) with freeze-dried PMB powders. Dynamic light scattering (DLS) was conducted on a Zetasizer Nano ZS (Malvern, USA) instrument to measure the size of the polymer in solution. Light obscuration (Accusizer 780A) was used to measure size distribution of MBs. Serum osmolarity was measured with a 5600 Vapro Pressure Osmometer (Wescor Vapro, USA).

Cryo-scanning electron microscopy (Cryo-SEM) samples were frozen in slush nitrogen (SN_2), then transferred into a MED 020 instrument fitted with a freeze fracture chamber. Samples were fractured at a temperature of -150°C and etched for 8 minutes at -95 ^{o}C and coated with 7 nm of PT/Pd. The sample was then transferred to a Zeiss Nvision 40 fitted with a Cryo stage using VCT 100 cryo transfer device and imaged at -150 ^{o}C.

Dissolution kinetics by sonography. A clinical ultrasound (Zonare Z One ultrasound system, Mindray North America, USA) was used for phantom imaging using a convex ultrasound transducer (C10-3). A 50 ml falcon tube was placed in front of the transducer, and both were immersed in degassed water. Phosphate buffer saline at various pHs were prepared between 9.0 to 3.8 by titrating sodium hydroxide or hydrochloric acid solution using a calibrated pH meter. To perform the phantom imaging, 20 ml of PBS buffer was added to the falcon tube with magnetic stirring at 500 rpm, then 0.1 ml of 10% PMBs were quickly pipetted to the center of the PBS solution that is in the field of view. The echo signals were continuously recorded throughout the dissolution process in M-mode (MI 0.9, frame rate 12 Hz). In a separate set of experiments, the falcon tube was initially placed outside (above) the beam path of the transducer and was quickly placed into the field-of-view at predetermined time interval to image after administering PMBs. All experiments were performed in triplicate.

Dissolution Kinetics by UV-vis spectroscopy (Cary 60, Agilent, USA). 2 ml of PBS that was pre-titrated to a pH between 9.0 to 3.8 was placed in a cuvette under, the absorbance at 600 nm was continuously measured at a rate of 10 Hz. After baseline measurement, 10 ul of 10% PMBs were quickly pipetted to the cuvette under stirring, the changes in absorbance were measured up to 10 minutes. All experiments were performed in triplicate.

Accelerated stability study. Fully oxygenated oPMB solutions (pO2 740 mmHg) were packaged in capped glass syringes or plastic tubes in a glovebox under oxygen environment. The syringes and tubes were then sealed with parafilm and placed in different desk incubators set at various temperatures (25 ^{o}C, 35 ^{o}C, 45 ^{o}C and 60 ^{o}C). Each incubator was purged with an oxygen headspace to maintain the oxygen environment and avoid potential oxygen loss from the syringes or tubes; in the future this would be addressed using a hermetically sealed plastic bag around the sealed syringe. oPMB samples were removed from the incubator only to record changes in foam height at select time intervals up to 30 days. The size of oPMBs stored under various temperatures was then measured by Accusizer to compare with their original size.

Supplementary Table 1 I Summary of doses of PMBs used in various animal studies.

	Figure	Gas content (oxygen fraction)	Gas fraction	Gas injection rate (ml of gas/kg/min)*	Total gas volume infused	Total volume infused
Rodent hemodynamic study	Fig. 3c-f	Air (21%O_2)	50% vol gas/vol foam	5 ml/kg/min	25 ml/kg	50 ml/kg
In vivo dissolution study by echo	Fig. 3g-l	Air (21%O_2) and 100% O_2	40% vol gas/vol foam	4 ml/kg/min; 8ml/kg/min; 12 ml/kg/min	4 ml/kg; 8 ml/kg; 12 ml/kg	10 ml/kg; 20 ml/kg; 30 ml/kg
Swine efficacy study	Fig. 4	100% O_2	35% vol gas/vol foam	4 ml/kg/min	12 ml/kg	33 ml/kg
Rodent safety	Fig. 5	100% O_2	40% vol gas/vol foam	1.3 ml/kg/min	13 ml/kg; 26 ml/kg	32 ml/kg; 64 ml/kg

*Gas injection rate was calculated as total foam volume injected/injection time (i.e., the time during which injections were actually occurring).

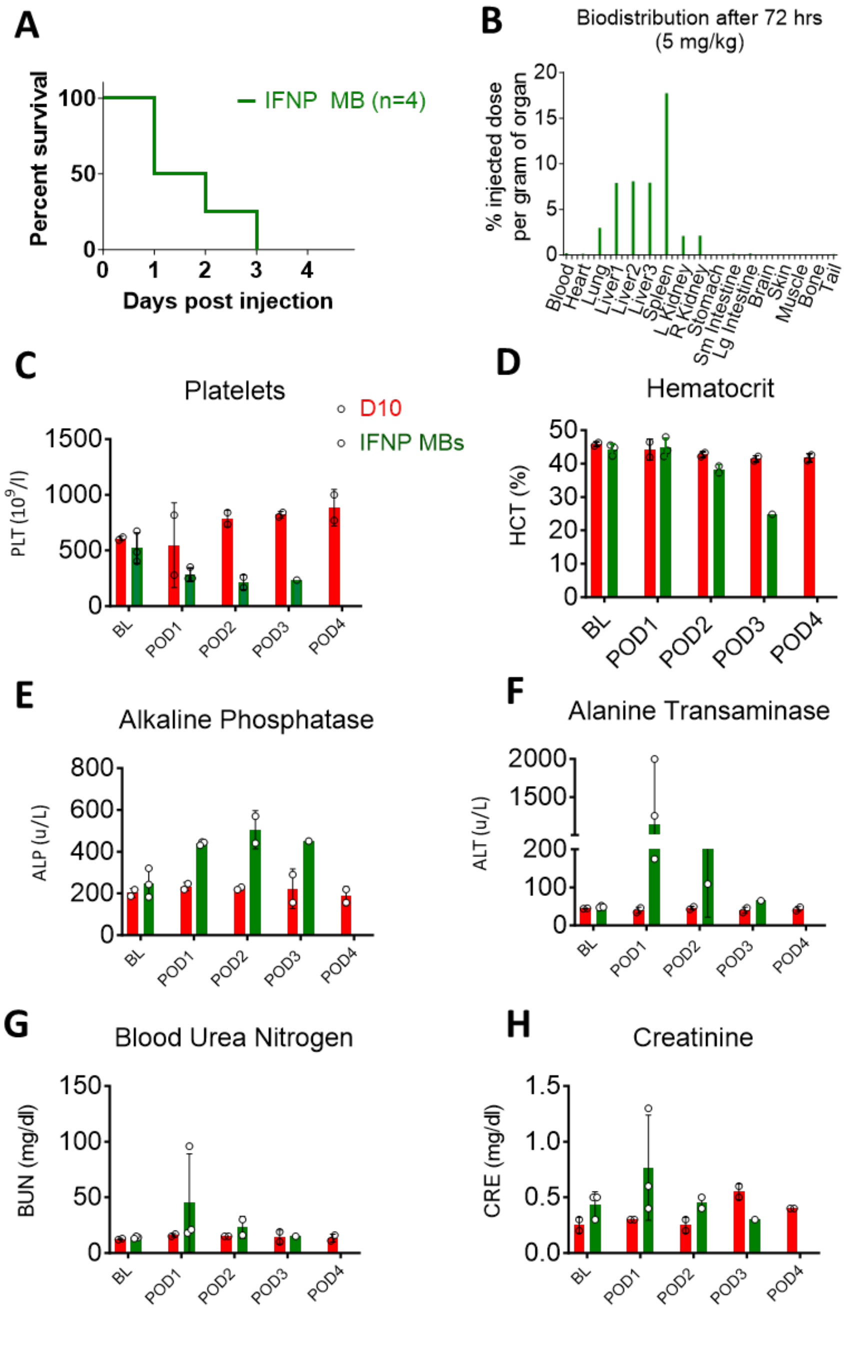

Supplementary Fig. 1 | Infusion of MB prepared via interfacial nanoprecipitation (IFNP MBs) resulted in organ injury and high mortality. The polymer was based on chemical modification of dextran precursor (25 kDa) with acetylation degree of 1.8 and succinylation degree of 0.6. **A**. Infusion of IFNP MBs (70%,18ml/kg) fabricated with a more hydrophobic polymer led to high mortality. **B**. A high degree of organ accumulation was found in rat biodistribution study of ^{64}Cu-labeled polymer. In comparison with control group receiving equal volume of D10, treated animals showed various clinical abnormalities including lower platelet counts (**C**) and lower hematocrit (**D**), and liver (**E, F**) and renal (**G, H**) toxicity. BL = baseline; POD = post-operative day.

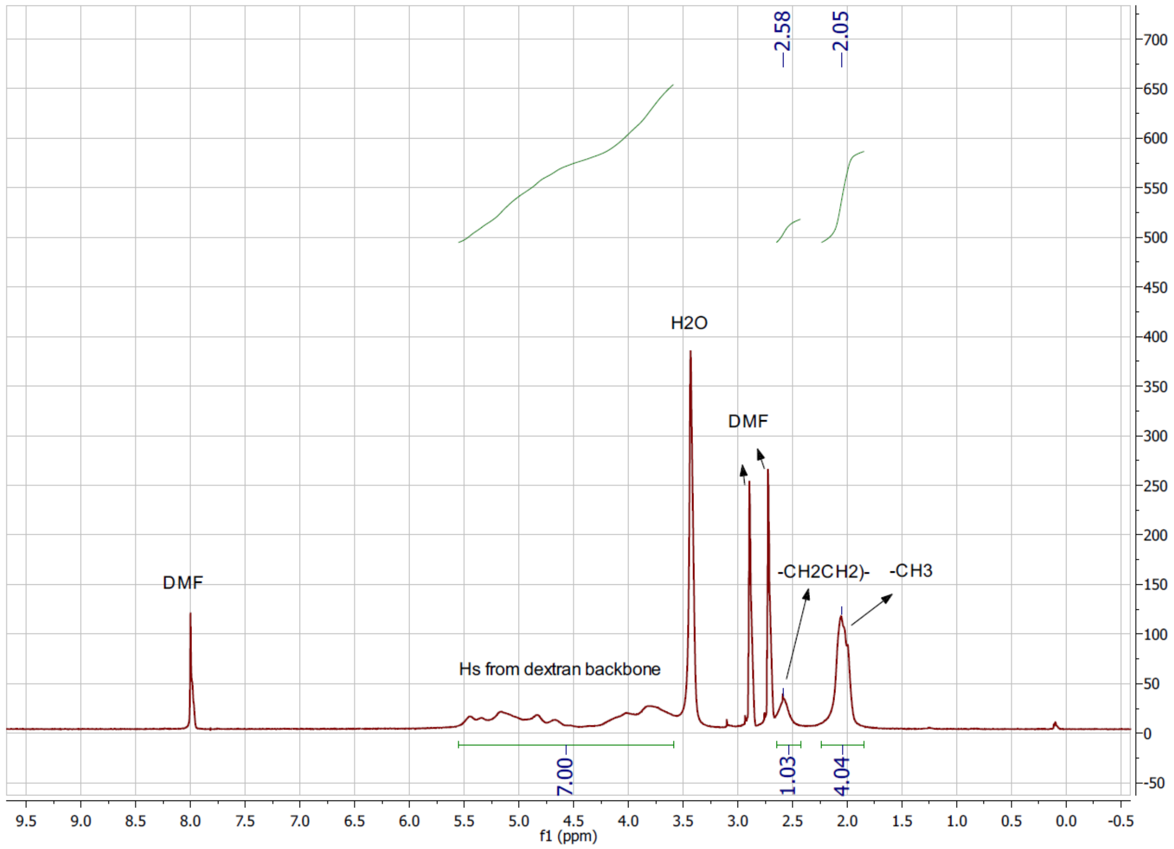

Supplementary Fig. 2 l 1H NMR of LmD polymer in DMF-d7, 400Hz. The calculation of degree of substitutions is as follows. Acetylation: 4.04 / 3 (-CH3) = 1.3; Succinylation: 1.03 / 4 (-CH2CH2-) = 0.26. The manufacture of MBs were successful with a variation in degree of substitution on tested polymers with acetylation between 1.2 and 2, and succinylation between 0.2 and 0.8.

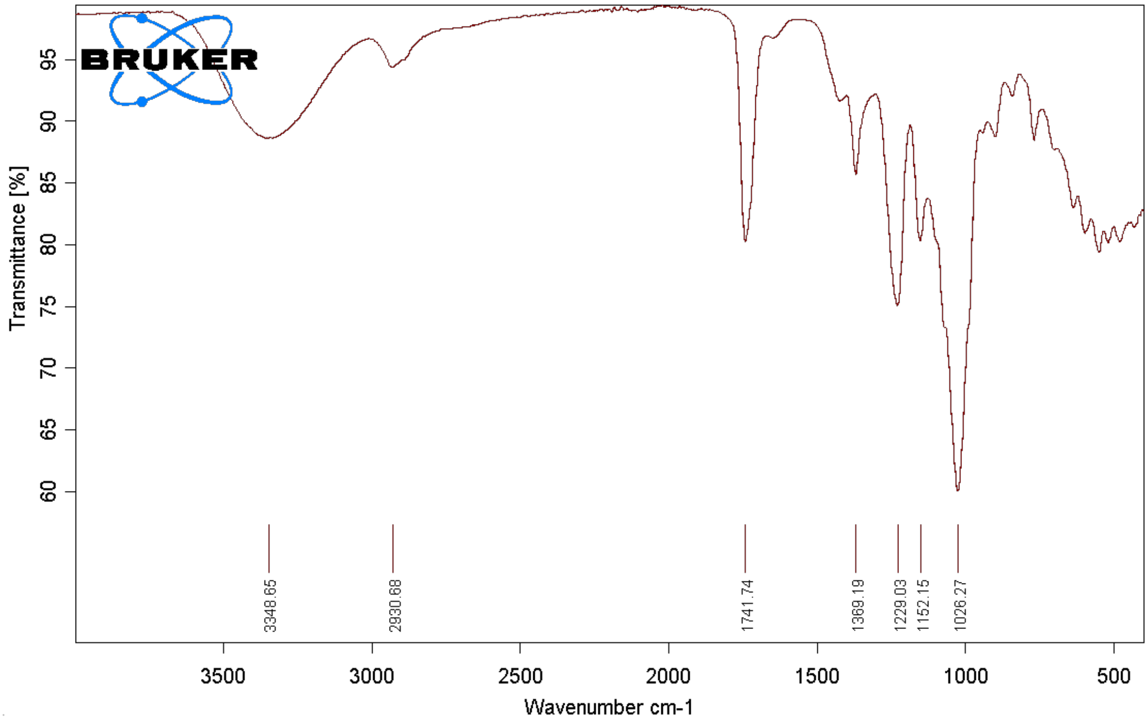

Supplementary Fig. 3 l IR spectrum of freeze-dried intact LmD PMB shells.

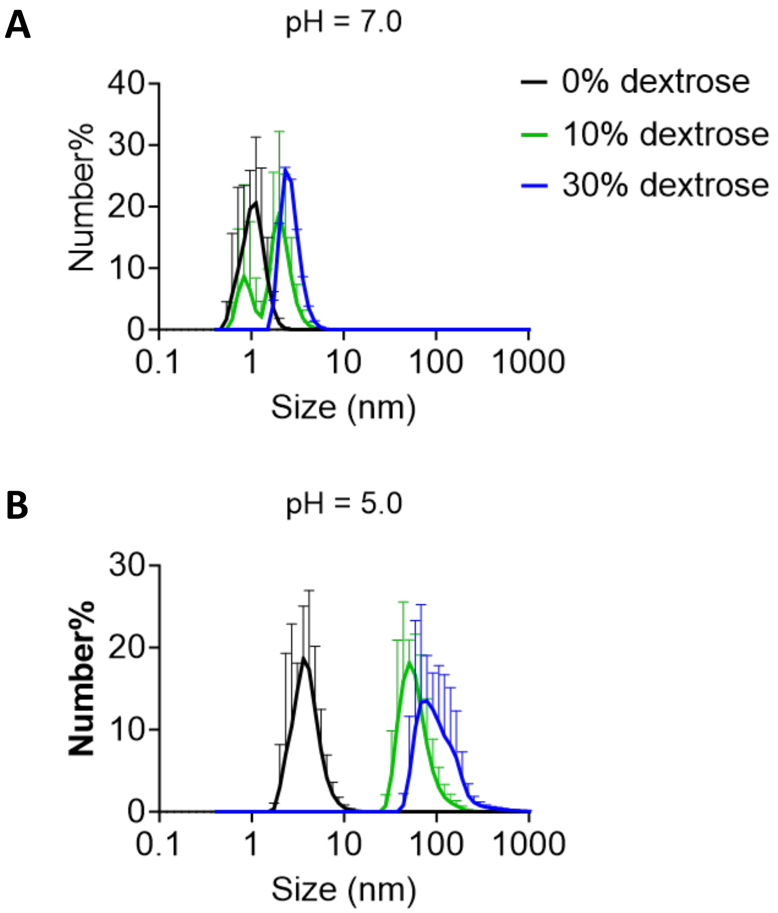

Supplementary Fig. 4 I Size of various LmD polymer (5mg/ml) aqueous solutions (with 0, 10 or 30% dextrose) at different pHs measured by dynamic light scattering (DLS). **A**. At pH 7, the sizes of polymer showed little dependence on the concentration of dextrose. **B**. At pH 5, where the polymer was partially protonated, increasing dextrose concentration led to observation of larger sizes, suggesting that higher concentrations of dextrose likely promoted the polymer aggregation at low pHs.

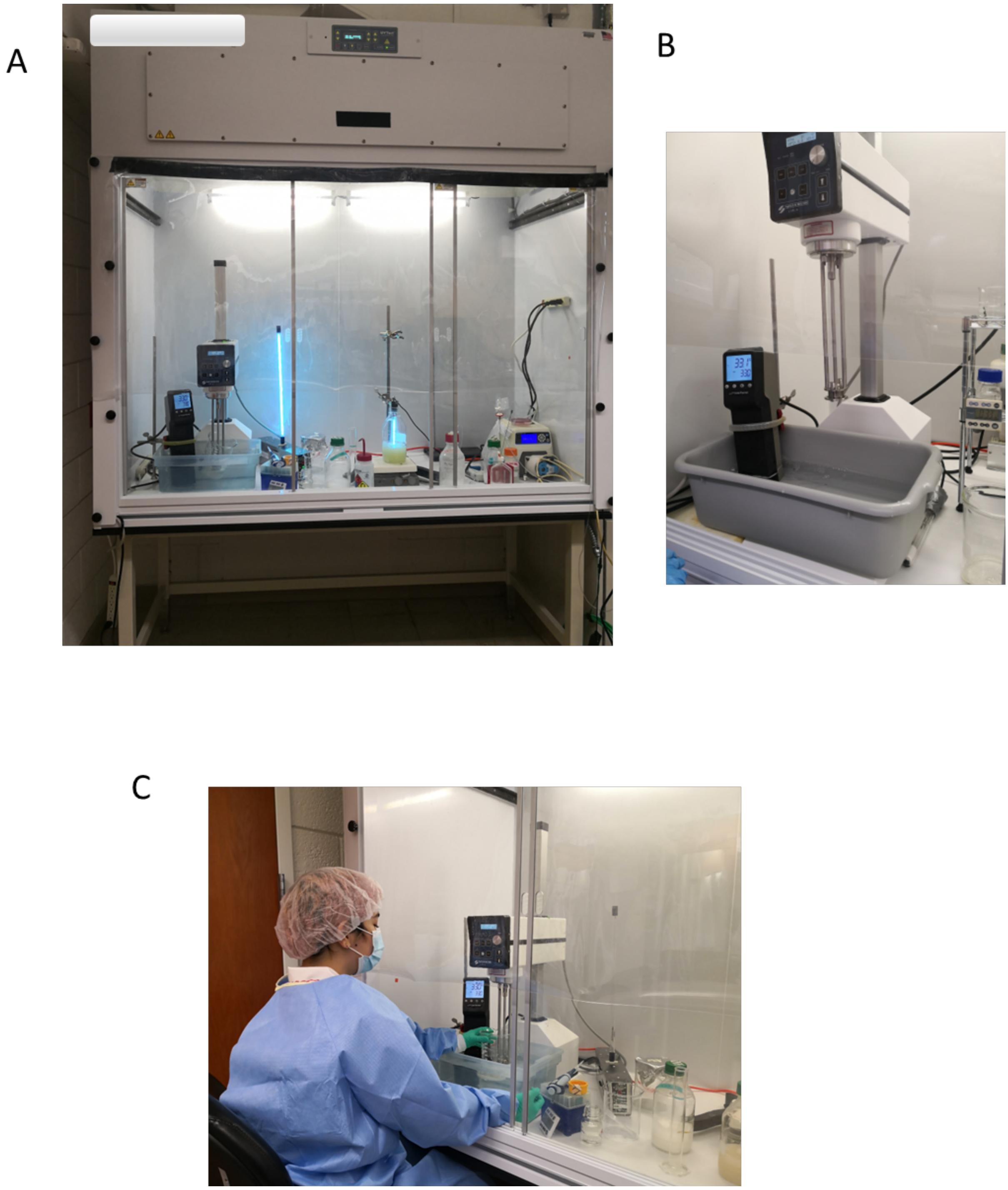

Supplementary Fig. 5 I Photos of manufacturing setup to produce LmD PMBs. **A**. The biosafety workstation of Vertical Laminar Flow Enclosure (dimension: WxDxH 2.4 x 0.9 x 1.8 m) that houses all sterile fabrication processes. **B**. The homogenization setup with heated water bath. **C**. A gowned researcher operating the homogenizer to prepare foam.

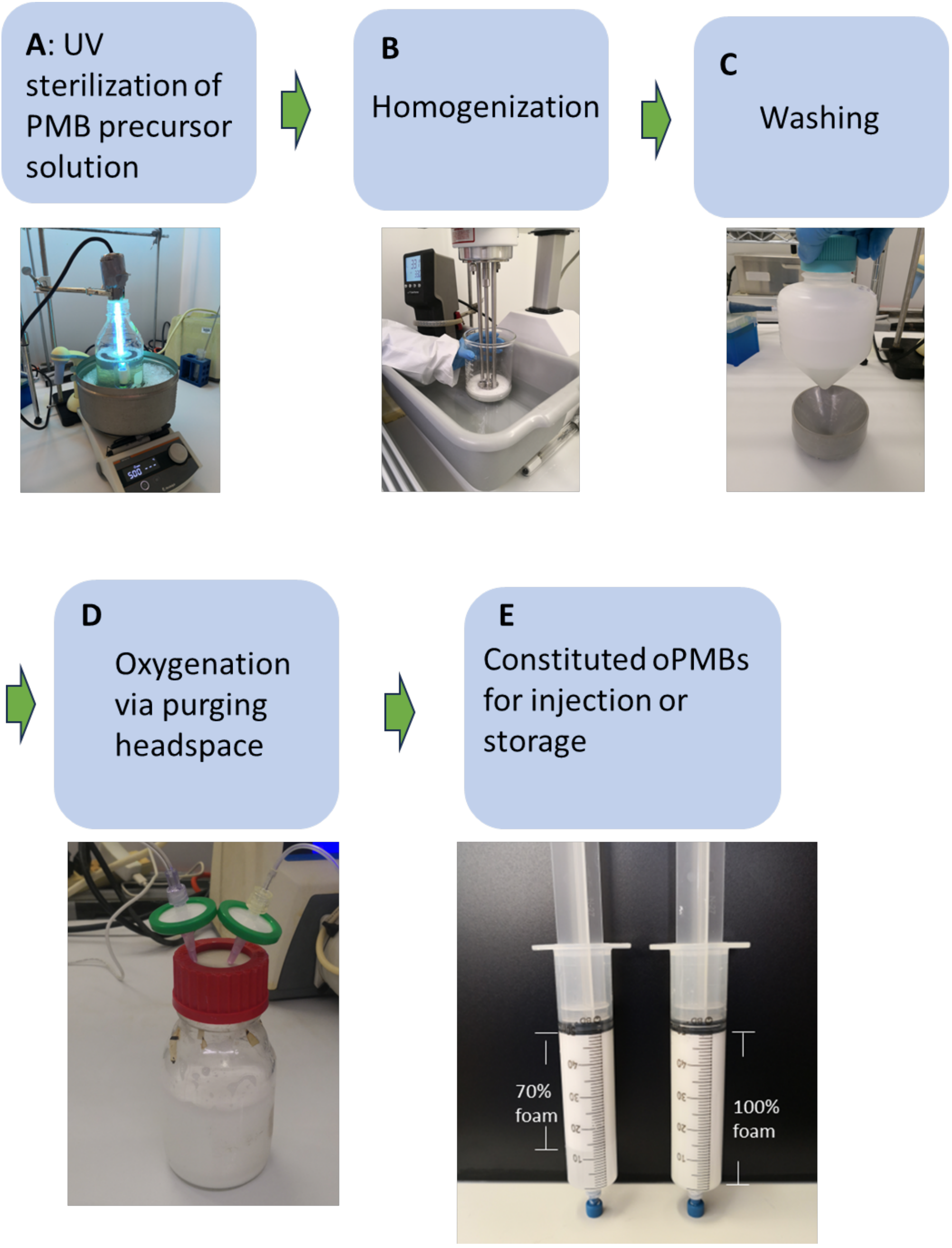

Supplementary Fig. 6 l Flowchart of LmD PMB fabrication processes. **A**. LmD solution was UV-sterilized on an ice bath. **B**. Homogenization was conducted in the water bath at 33 ^{o}C. **C**. The freshly prepared foam was transferred to conical containers, where the foam was concentrated on the top and the bottom solution removed by a pump via a long needle and repeated washed with D10. **D**. Air-filled PMBs (aPMBs) were oxygenated via passive purging with humidified pure oxygen gas. **E**. The final constituted oxygen filled PMBs (oPMBs) in syringe. Left showed a 70 v/v% foam (40% gas content), the right showed a 100% foam (60% gas content).

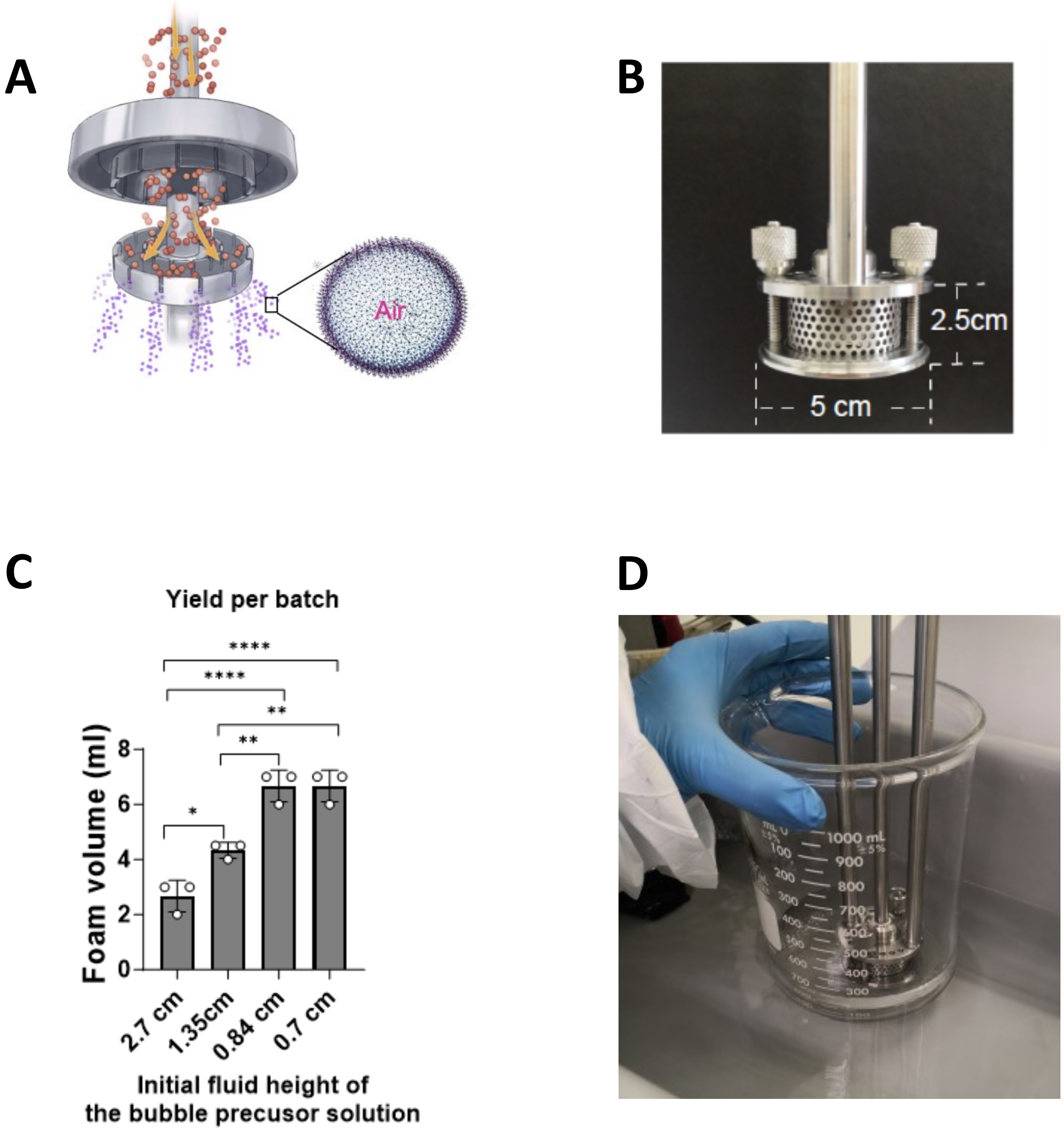

Supplementary Fig. 7 I Batch volume optimization. **A**. Cartoon illustration of foam generation via mixing head that breaks up large bubbles into small ones. **B**. The dimension of mixing head used in the current work. C. Initial fluid height and volume affected the batch product (foam) yield. **D**. The batch volume used in current study, where the homogenizer head was also partially immersed. Optimal foam generation requires the mixing of liquid with air. Therefore, the optimal position of mixing head is at the fluid/air interface. As shown in **C**, increasing the initial fluid height decreased the foam yield likely due to less effective mixing with air, as the rotor was mostly submerged in liquid.

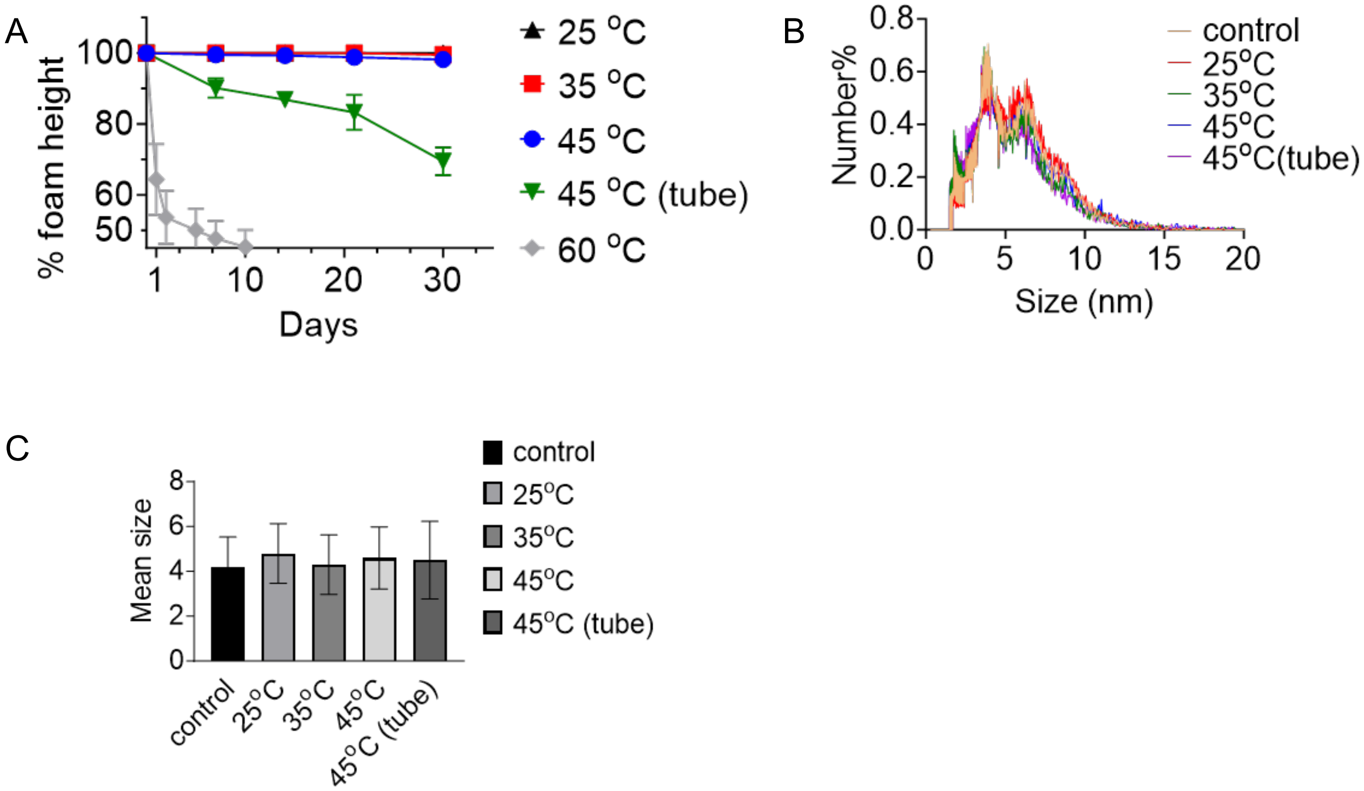

Supplementary Fig. 8 I Accelerated aging testing of oPMBs in storage showed that oPMBs stored in capped syringes are stable at 25°C, 35°C and 45°C for 30 days without showing size changes or loss of foam volume (i.e., gas content). **A**, Changes in foam height/volume of oPMBs over time when stored at different temperatures. Except where noted, oPMBs were stored in capped syringes. Syringe-stored oPMBs are stable in all temperatures except at 60°C. oPMBs stored in plastic tubes at 45°C also showed moderate loss of product, likely due to surface drying effects. **B**, the size distribution and mean diameter (**C**) of oPMB foams did not change over time. (All measurements were collected as biological triplicates)

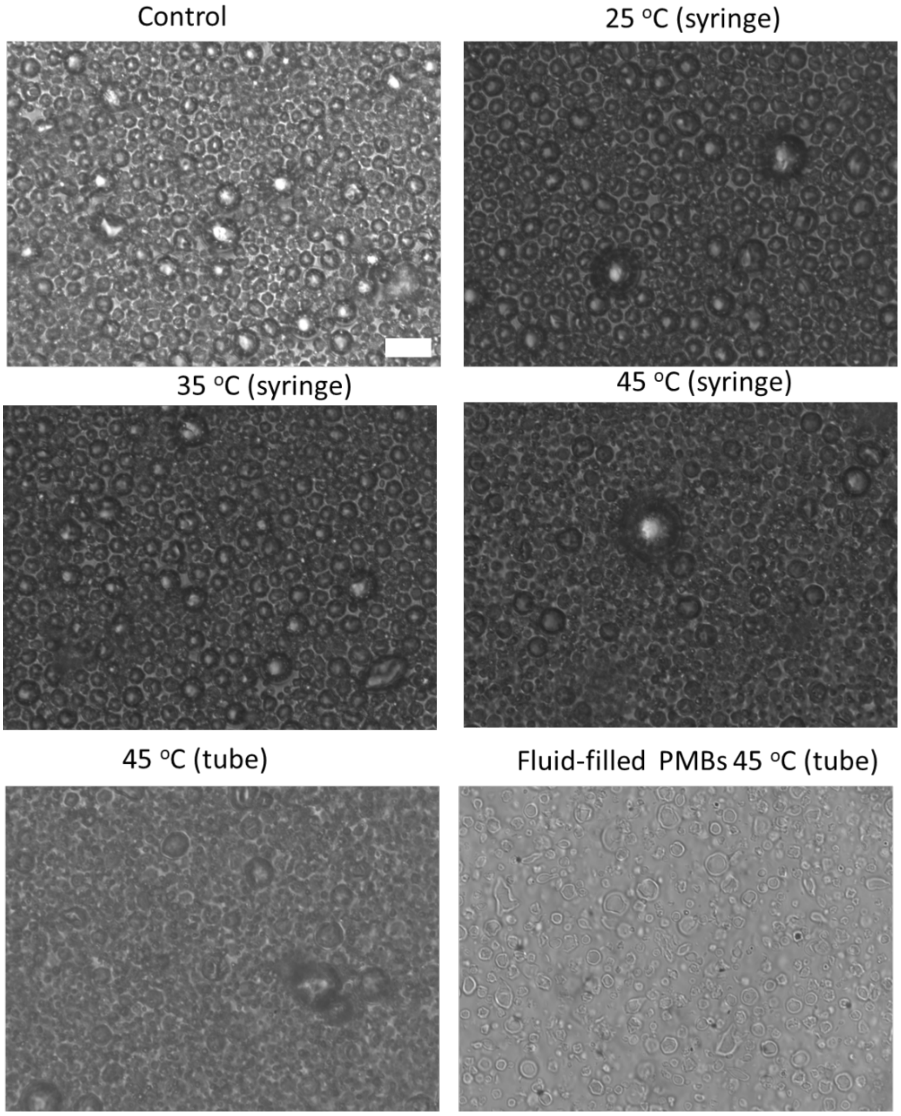

Supplementary Fig. 9 l Optical microscopy of oPMBs after 30-day storage at various temperatures as in **Supplementary Fig. 8**. No observable size or morphological changes were seen for oPMBs stored in glass syringes. Despite a moderate loss of foam when stored in plastic tube at 45°C, the remaining foam (at the top of the solution) did not change size or morphology (Bottom left image). Instead, product loss was due to fluid infiltration into gas core, leading to fluid-filled particles that separate from the top foam to sediment at the bottom (Bottom right figure). These observations also highlight that, unlike LOMs which undergo Ostwald ripening and consolidate into larger bubbles, oPMBs contain a hard-shell and do not suffer from changes in size distribution in storage. (Scale bar 10µm)

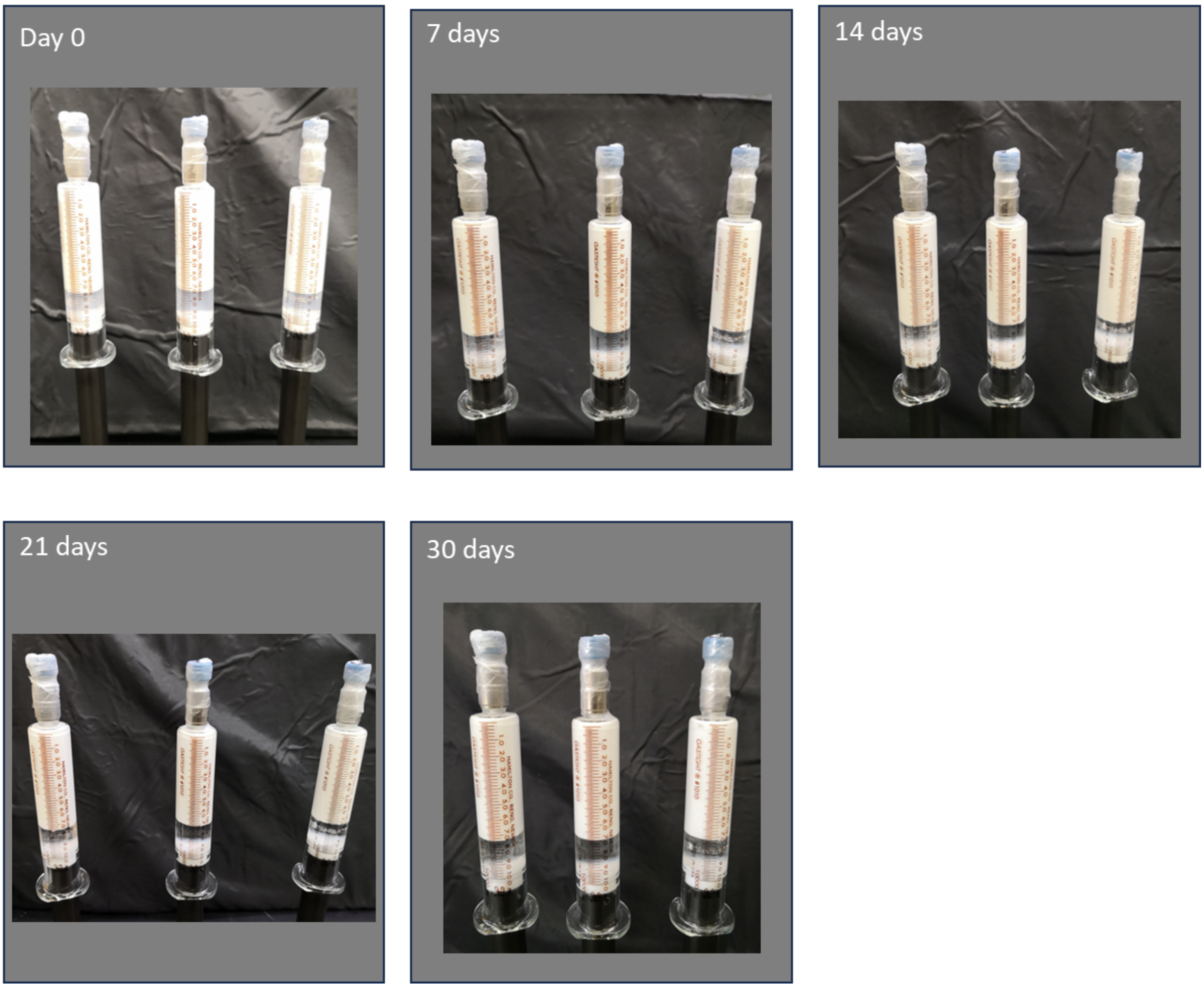

Supplementary Fig. 10 l Photos of oPMBs stored in glass syringes at 45°C taken at the timepoints shown (as in **Supplementary Fig. 8**).

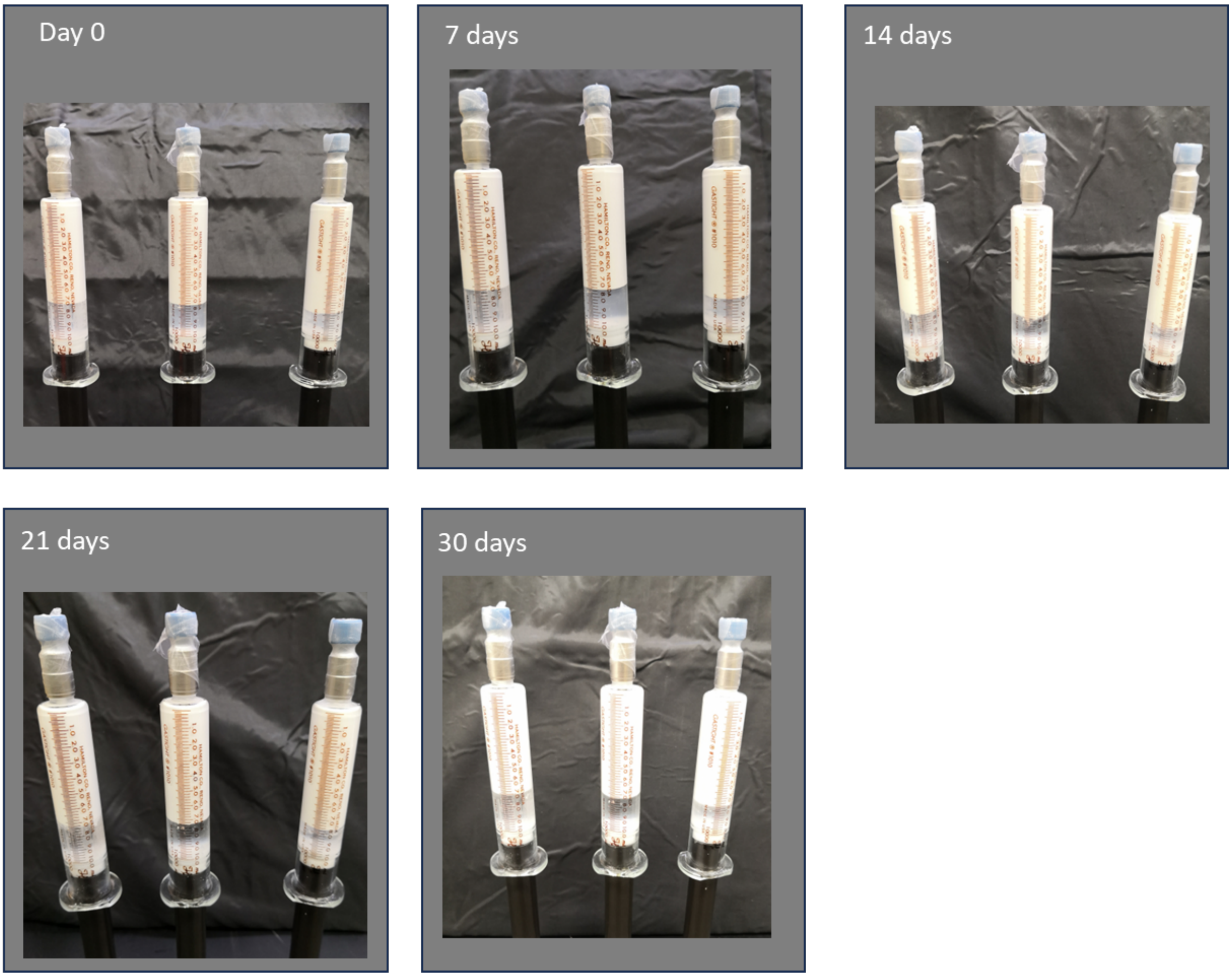

Supplementary Fig. 11 | Photos of oPMBs stored in glass syringes at 35°C taken at different timepoint (as in **Supplementary Fig. 8**).

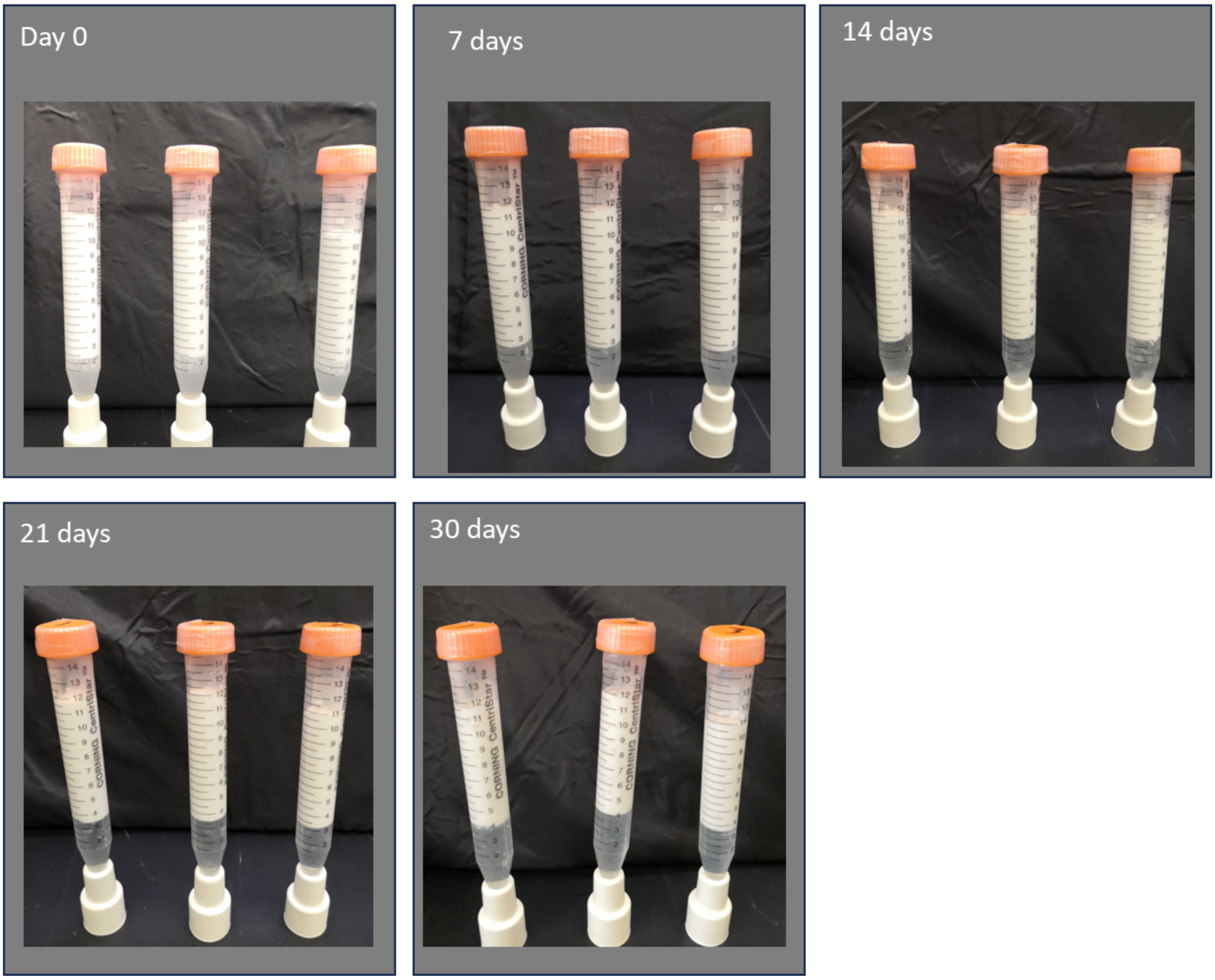

Supplementary Fig. 12 l Photos of oPMBs stored in plastic syringes at 45°C taken at the timepoints shown (as in **Supplementary Fig. 8**). Moderate decrements in foam volume were observed over time.

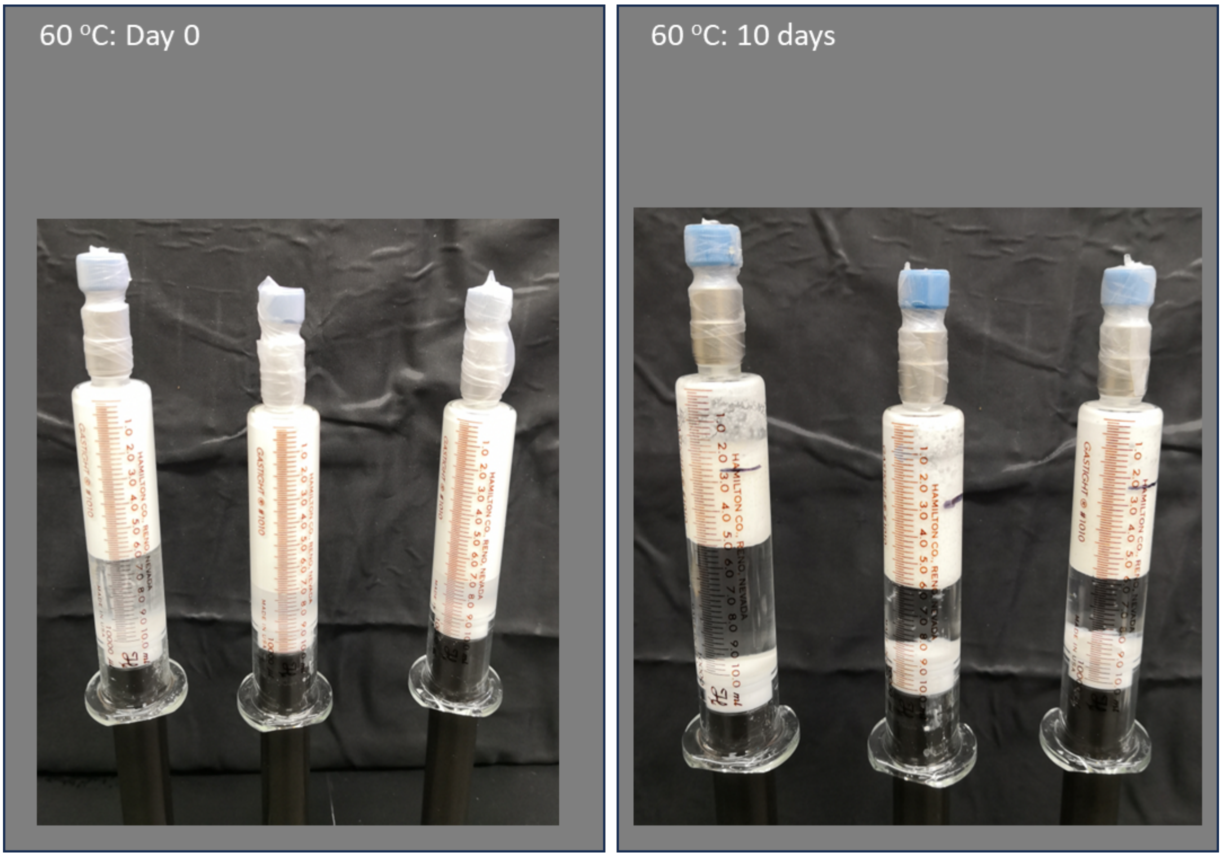

Supplementary Fig. 13 l Photos of oPMBs stored in plastic syringes at 60°C taken at the timepoints shown (as in **Supplementary Fig. 8**). Significant loss of foam volume was observed, indicated by the decreased total foam height as well as the development of large gas pocket at the top of syringe (marked by a black line to assist viewing. Note that on day 0, all syringes were fully filled with oPMB solution with no visible headspace.

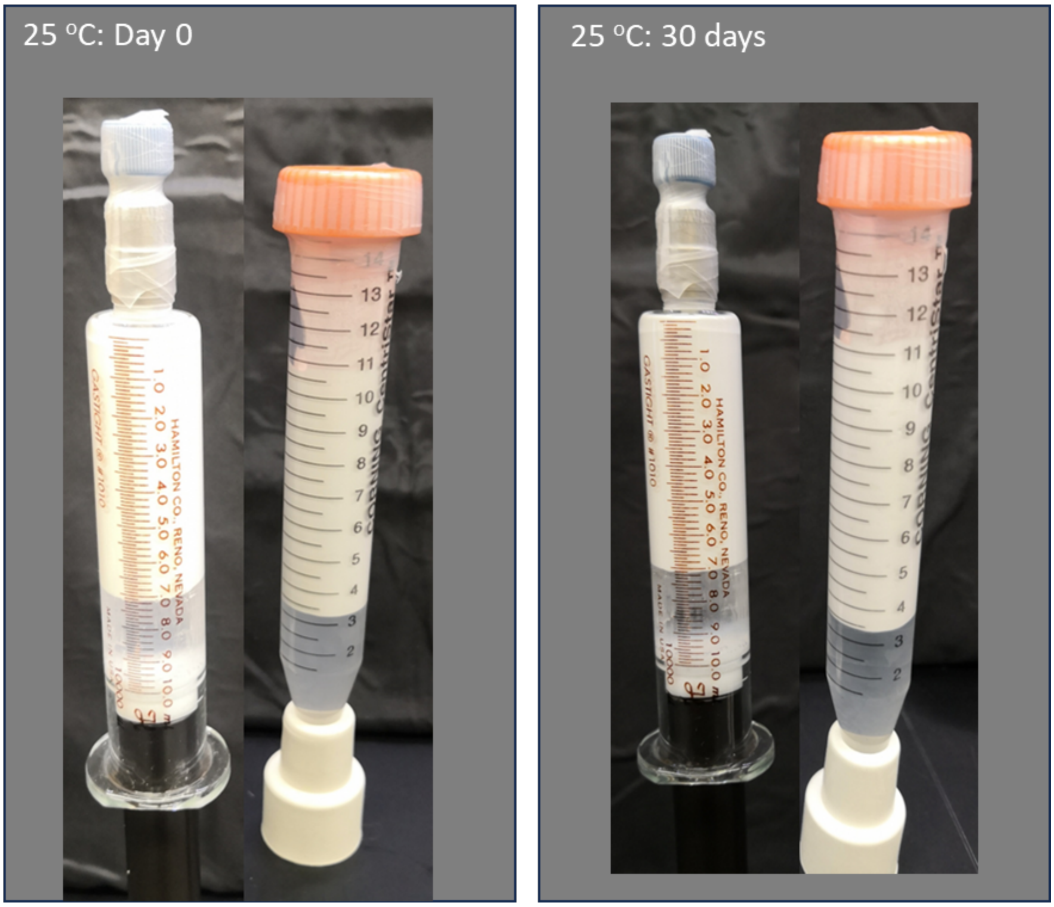

Supplementary Fig. 14 l Photos of oPMBs stored at 25°C (as in **Supplementary Fig. 8**) showed that they are stable both in glass syringes and plastic tubes.

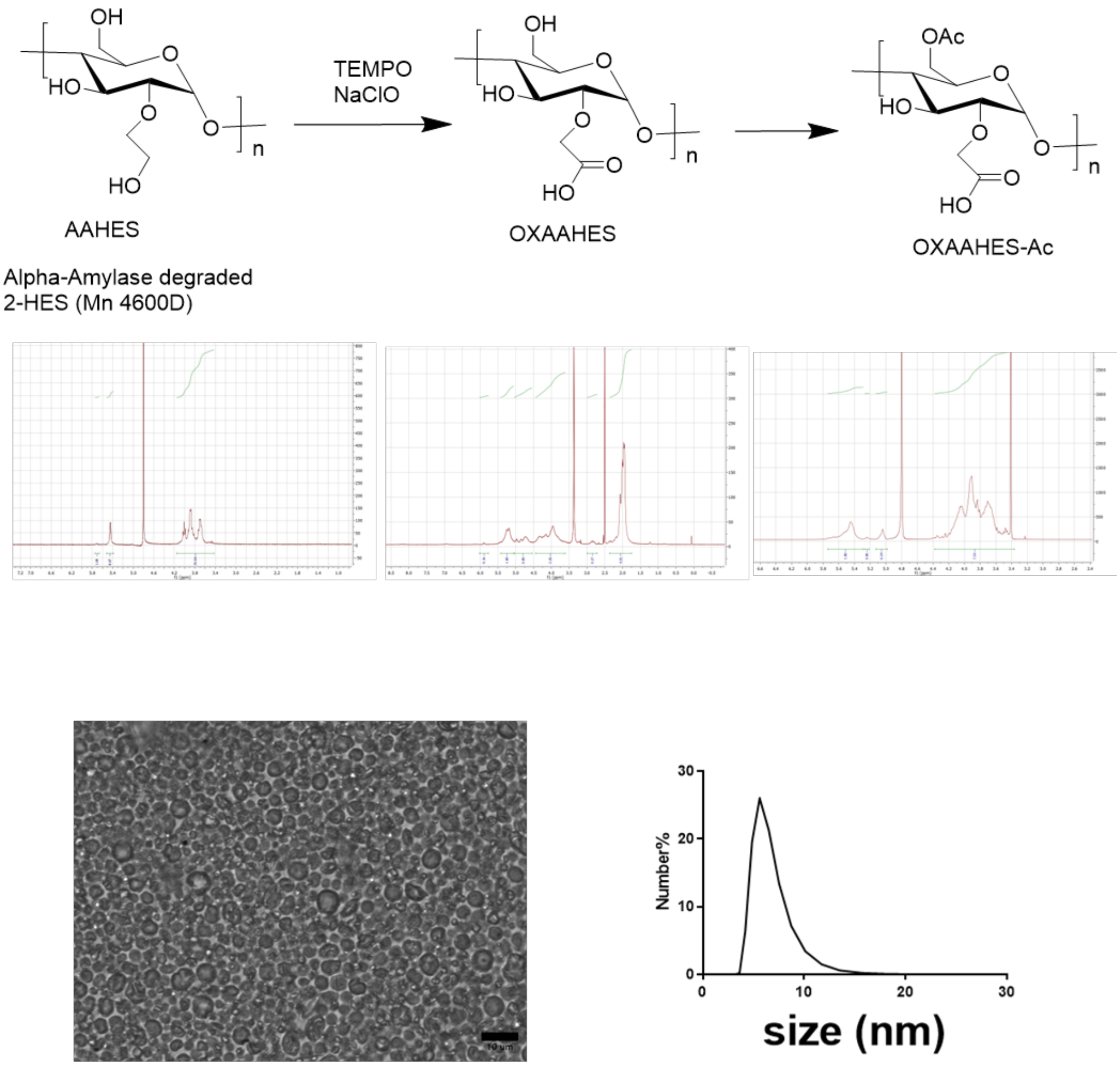

Supplementary Fig. 15 l Preparation of PMBs via interfacial crosslinking of modified hydroxyethyl starch, an optical microscope image showing the final product (scale bar 10 μm), and the OXAAHES-Ac PMB can rapidly dissolve in PBS (pH 7.2) to revert to soluble polymer with a mean hydrodynamic radius smaller than 10 nm.

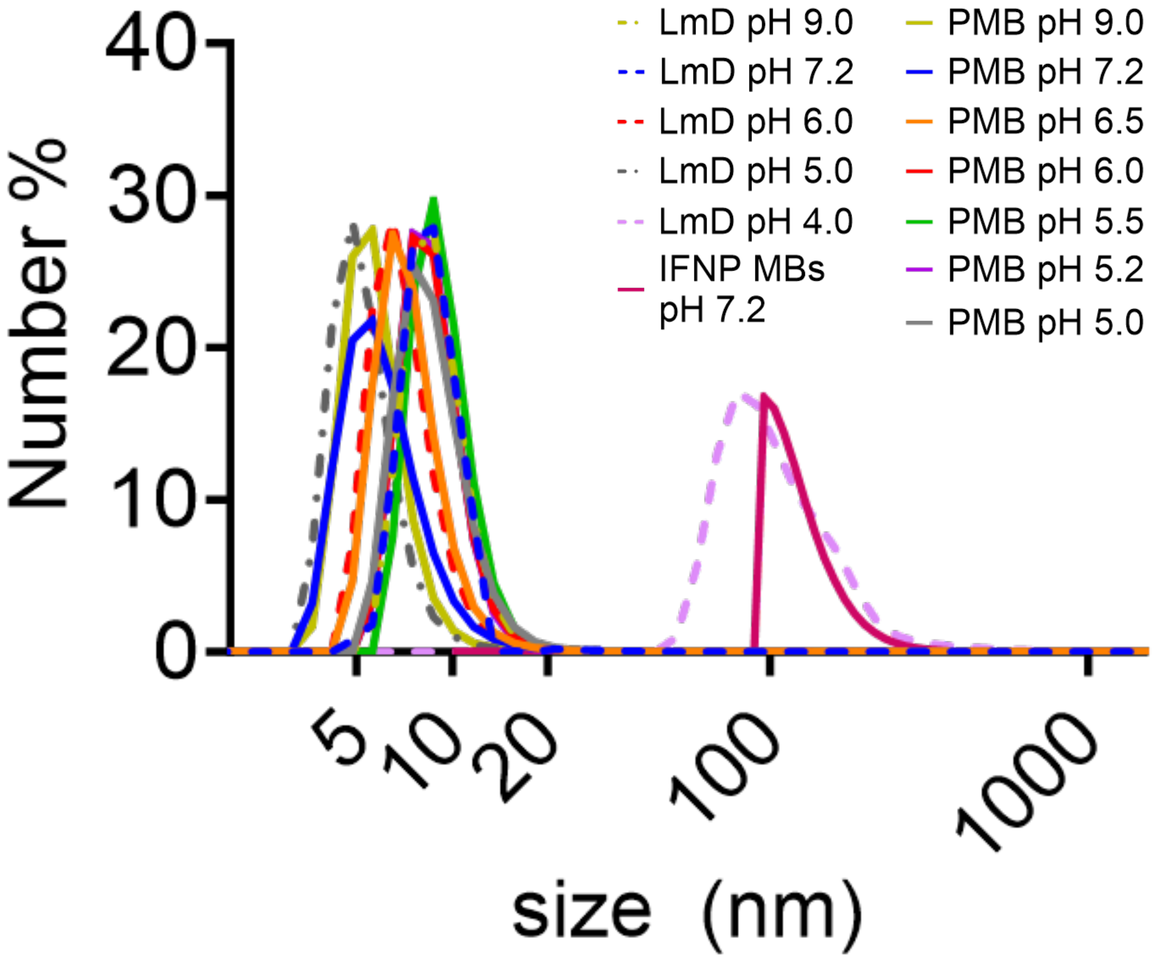

Supplementary Fig. 16 l Enlarged view of **Fig 2a**.

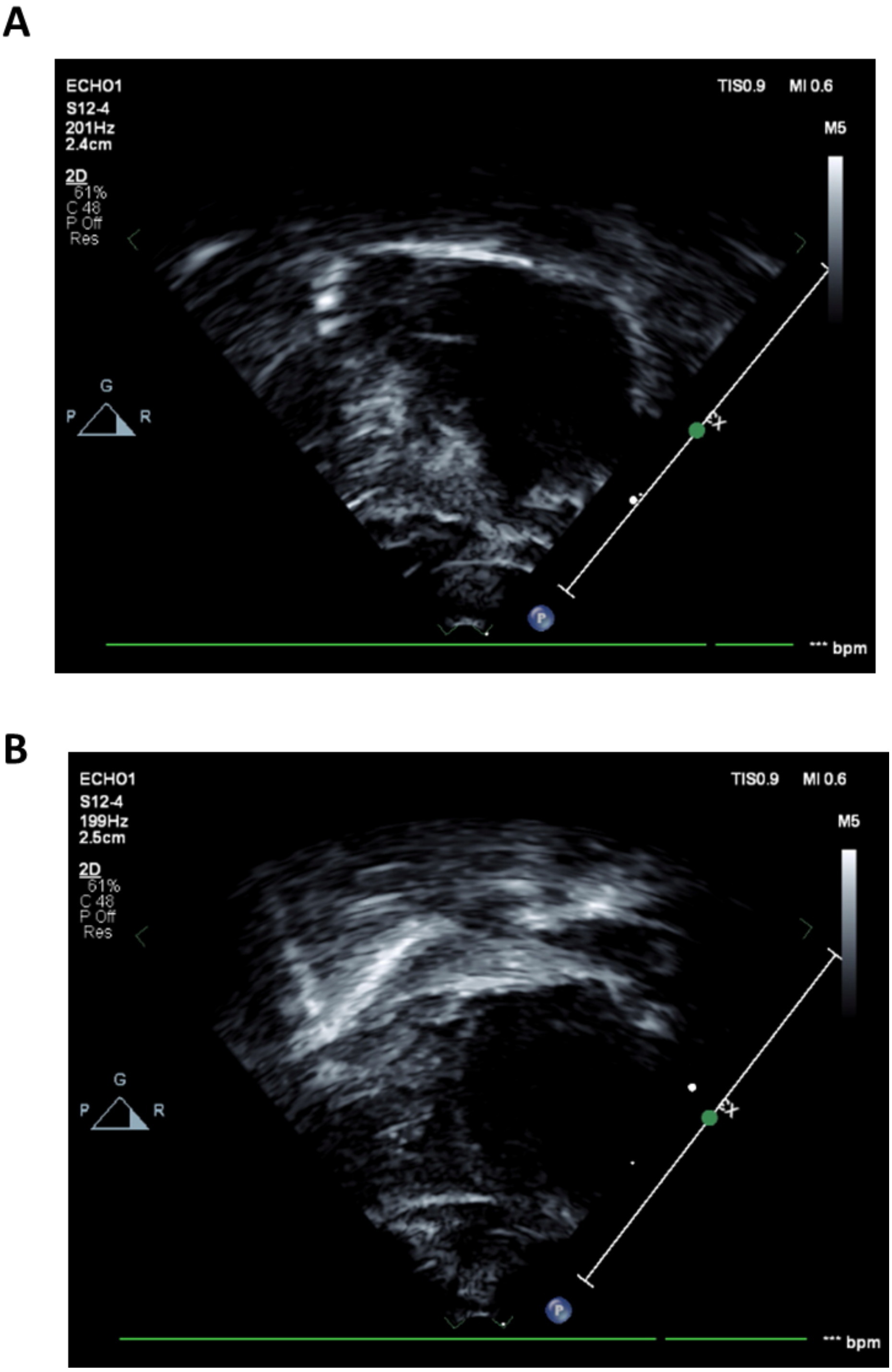

Supplementary Fig. 17 l Representative images from echocardiography of rat hearts receiving oPMBs at **A**: 8 mL O2 /kg/min and **B**:12 mL O2 /kg/min, showing no visible bubbles or opacification in the left heart.

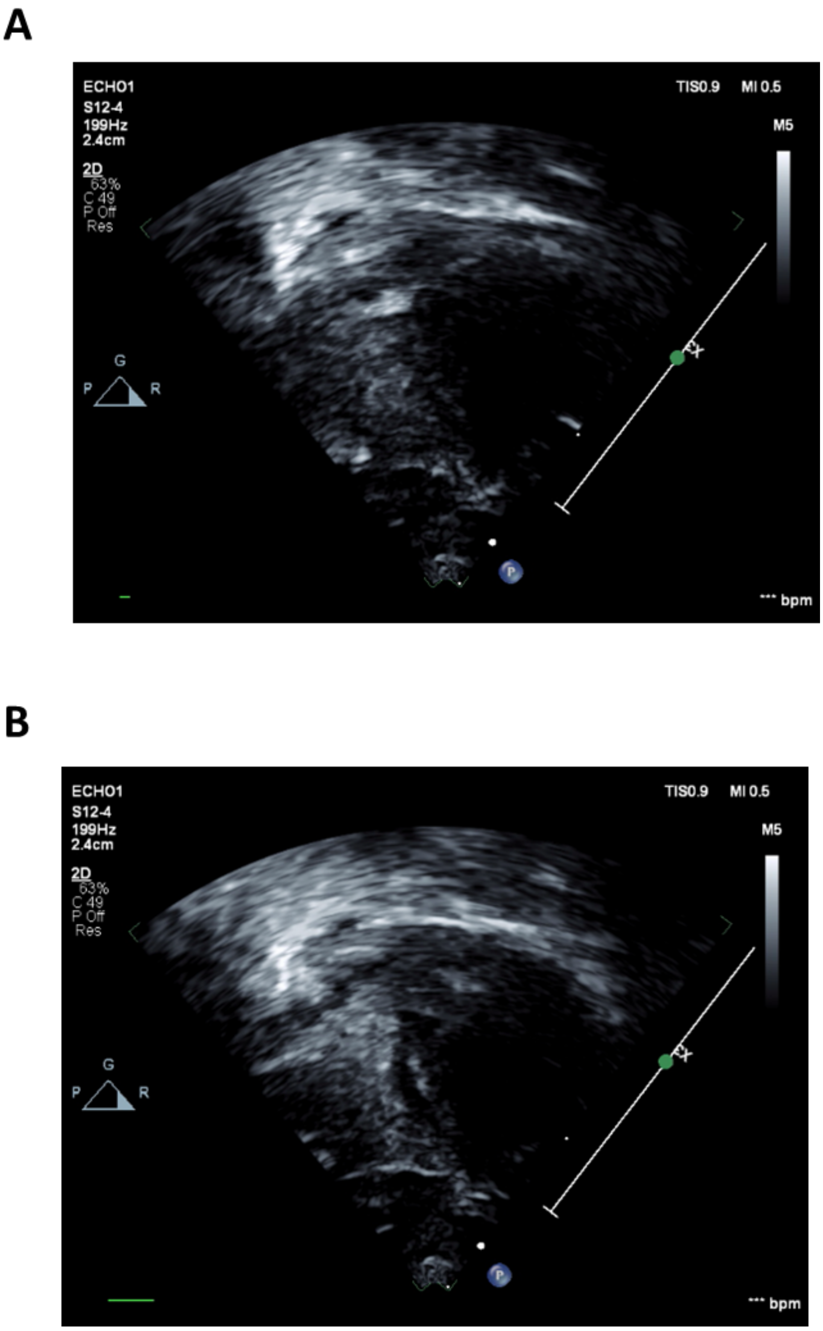

Supplementary Fig. 18 l Representative images from echocardiography of rat hearts receiving aPMBs at different infusion rate of gas volume. **A**: 8 mL air /kg/min and **B** 12 mL air /kg/min, with minimal opacification detected in the left chambers.

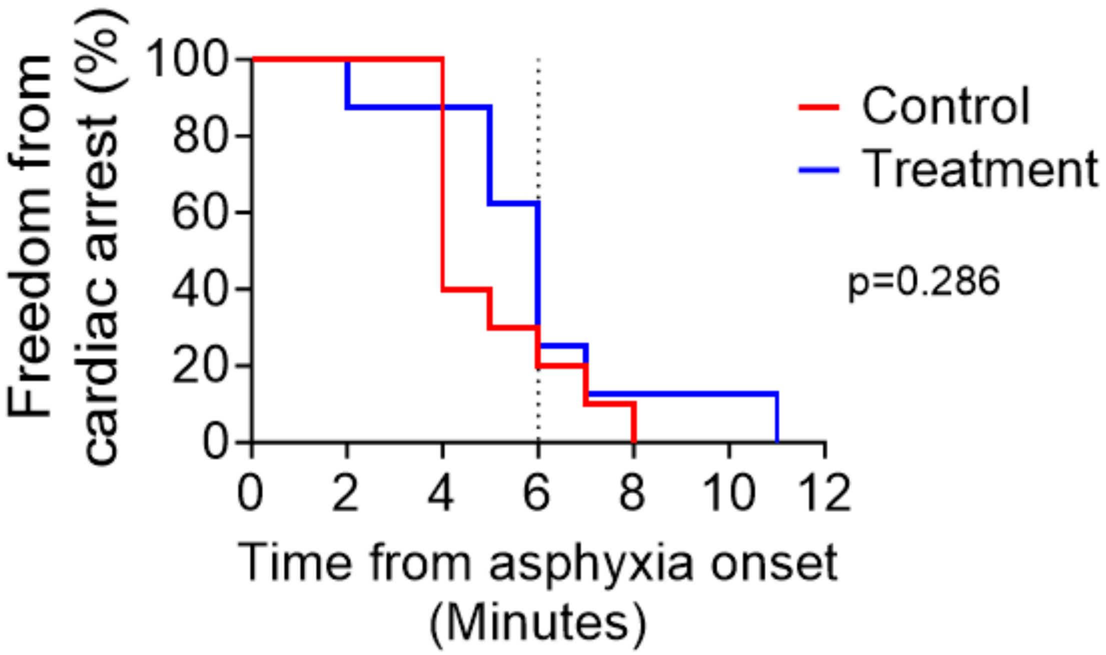

Supplementary Fig. 19 l Onset time of cardiac arrest during asphyxial model showing no significant difference between two groups. (Freedom from cardiac arrest was defined as %number of animals that had not experienced cardiac arrest at any point of time)

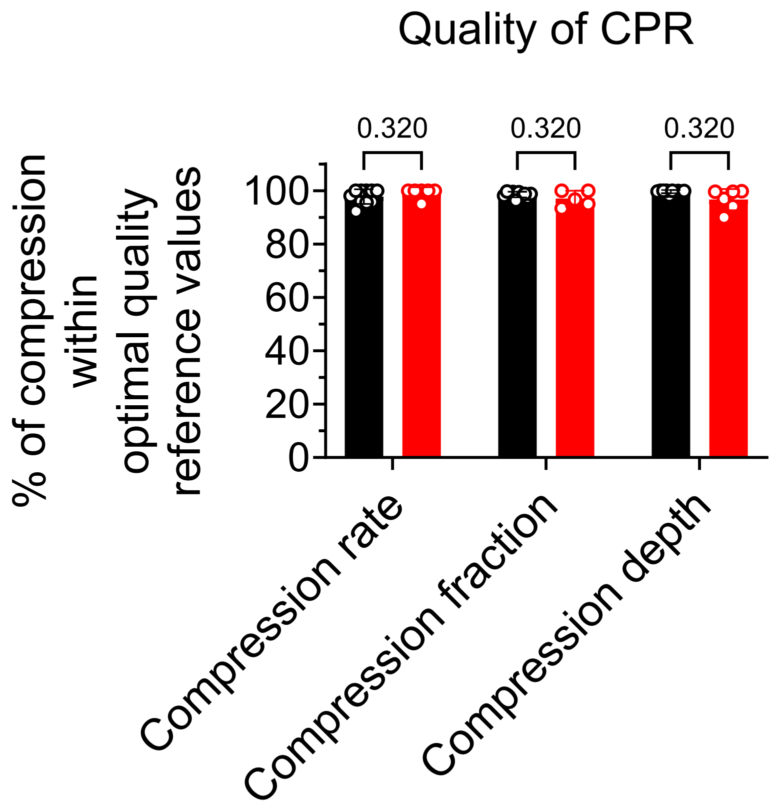

Supplementary Fig. 20 l CPR quality during asphyxia efficacy model was similar between treatment (red) and control (black) groups. Data presented as mean ± SD. Statistical analysis by Student's t-test with p values shown in the graph.

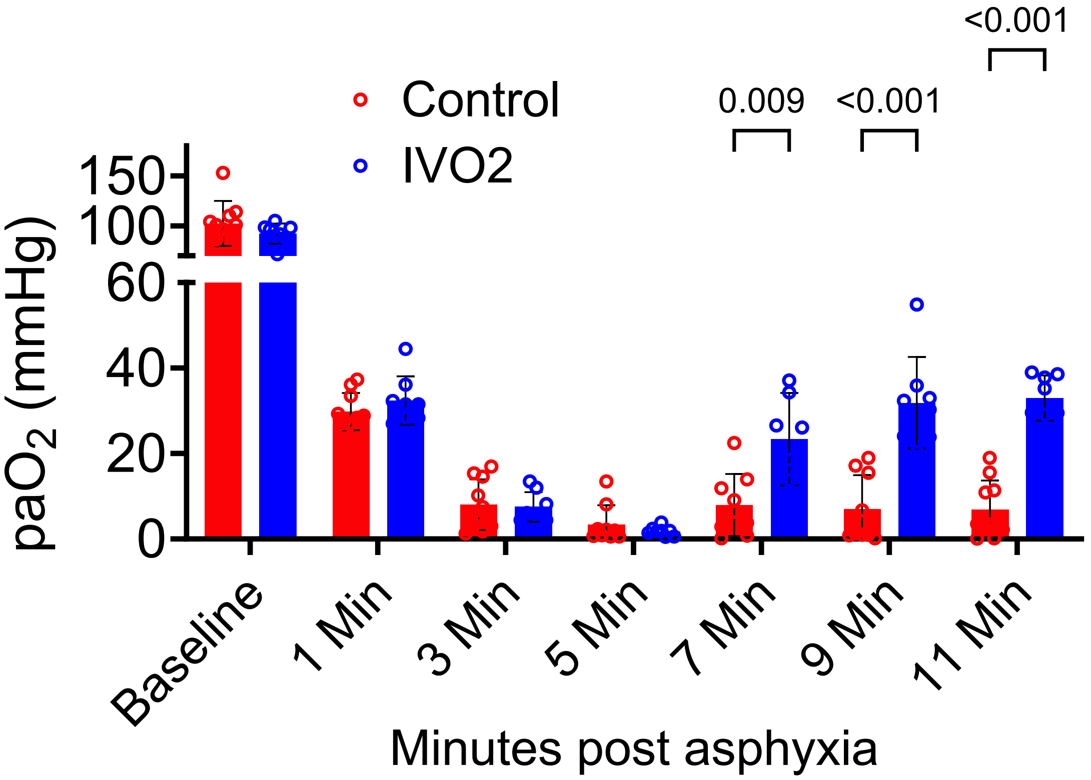

Supplementary Fig. 21 l The arterial partial pressure of oxygen (PaO_2) was significantly higher at 7, 9, 11 minutes in the IVO2-treated animals than those in the control group. Data presented as mean ± SD; red = control, blue = oPMB-treated swine. Statistical analysis by a two-way ANOVA with Tukey's multiple comparisons post-test.

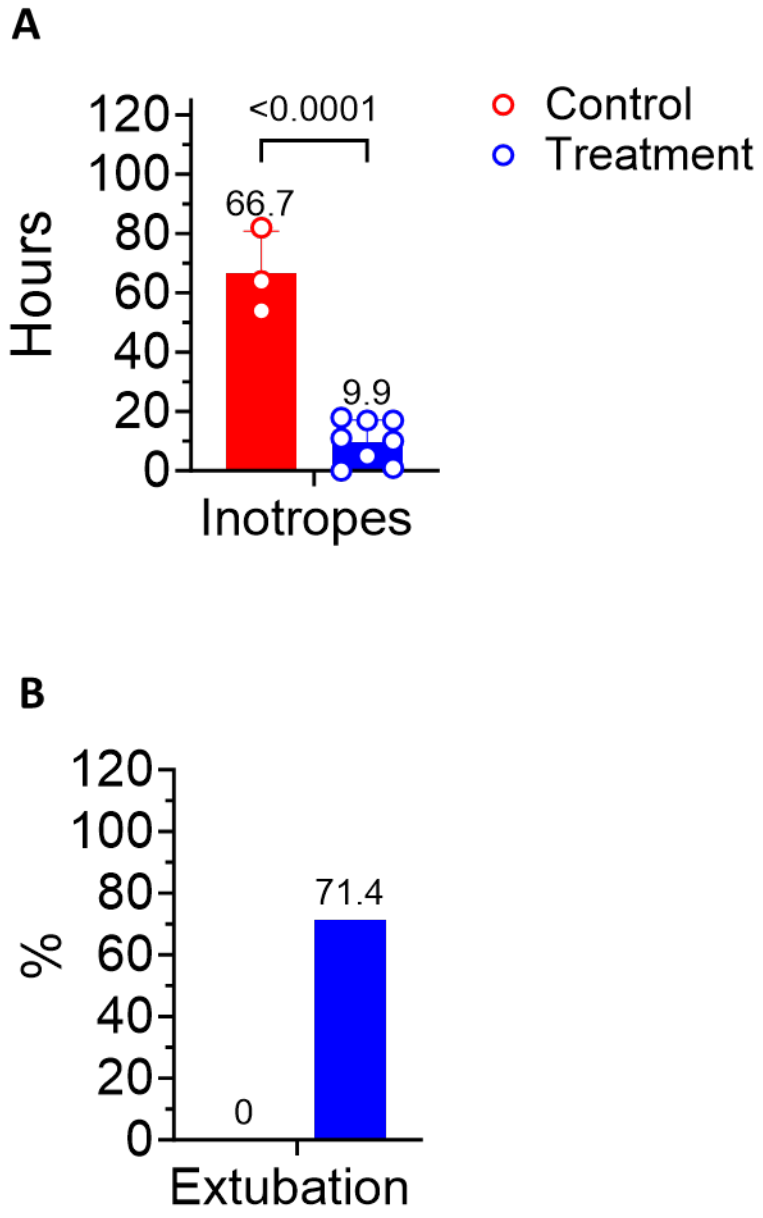

Supplementary Fig. 22 l (**A**) Surviving animals from treatment group received significantly less inotrope support than those from control group. Data presented as mean ± SD. Statistical analysis performed by Student's t-test with p values shown in the graphs. **(B**) None of surviving animals from control group were successfully extubated (0%), 5 out of 7 surviving animals from treatment group were successfully extubated (71.4%).

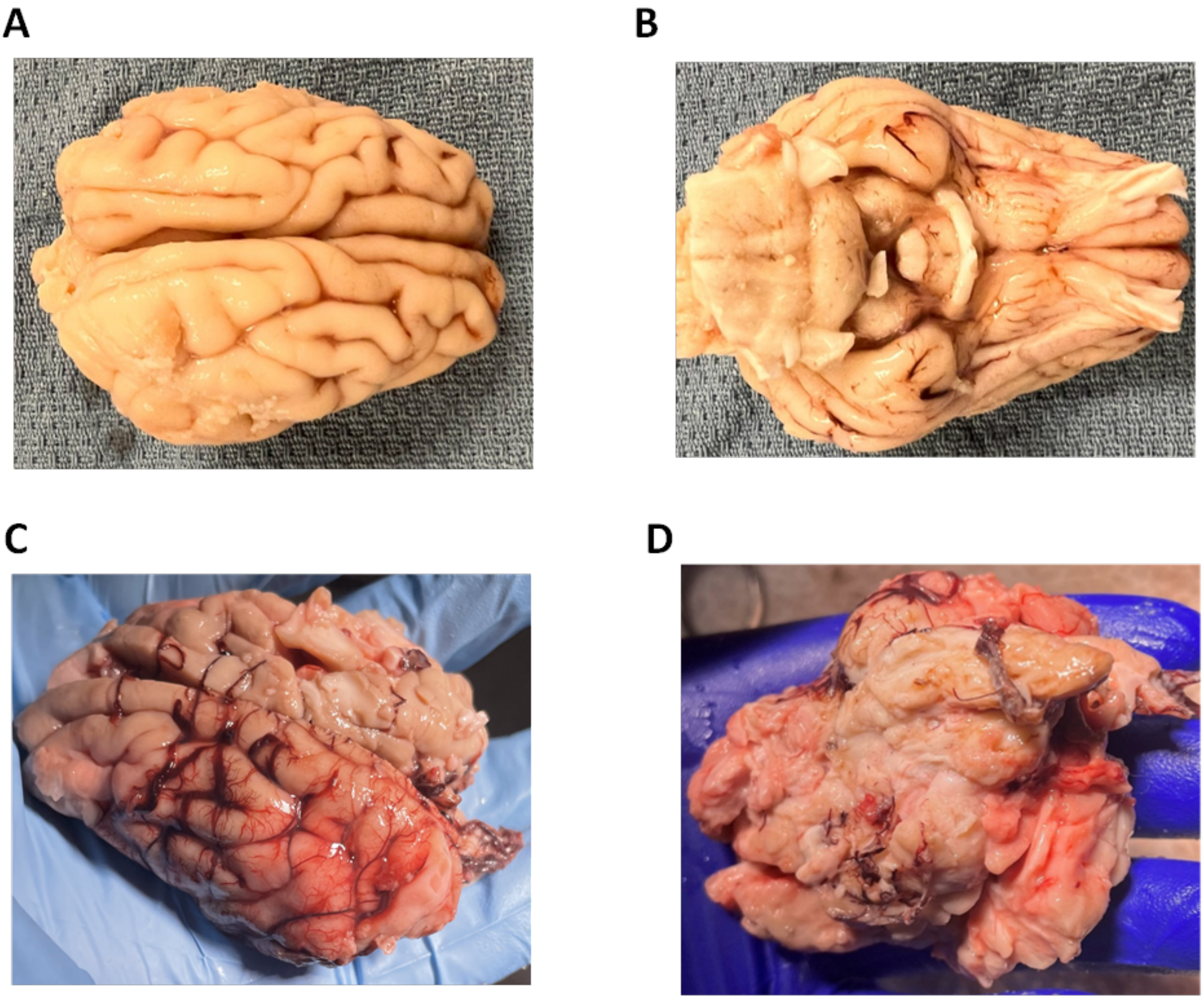

Supplementary Fig. 23 l Photos (enlarged) of pig brains taken at autopsy after survival experiments and brain MRI on day 4. **A, B**: representative photos from the treatment groups, showing a well-preserved brain. **C, D**: representative photos from the control group, showing swollen friable brain tissue with severe maceration of the specimen's ventral surface. **A, C**: dorsal; **B, D**: ventral.

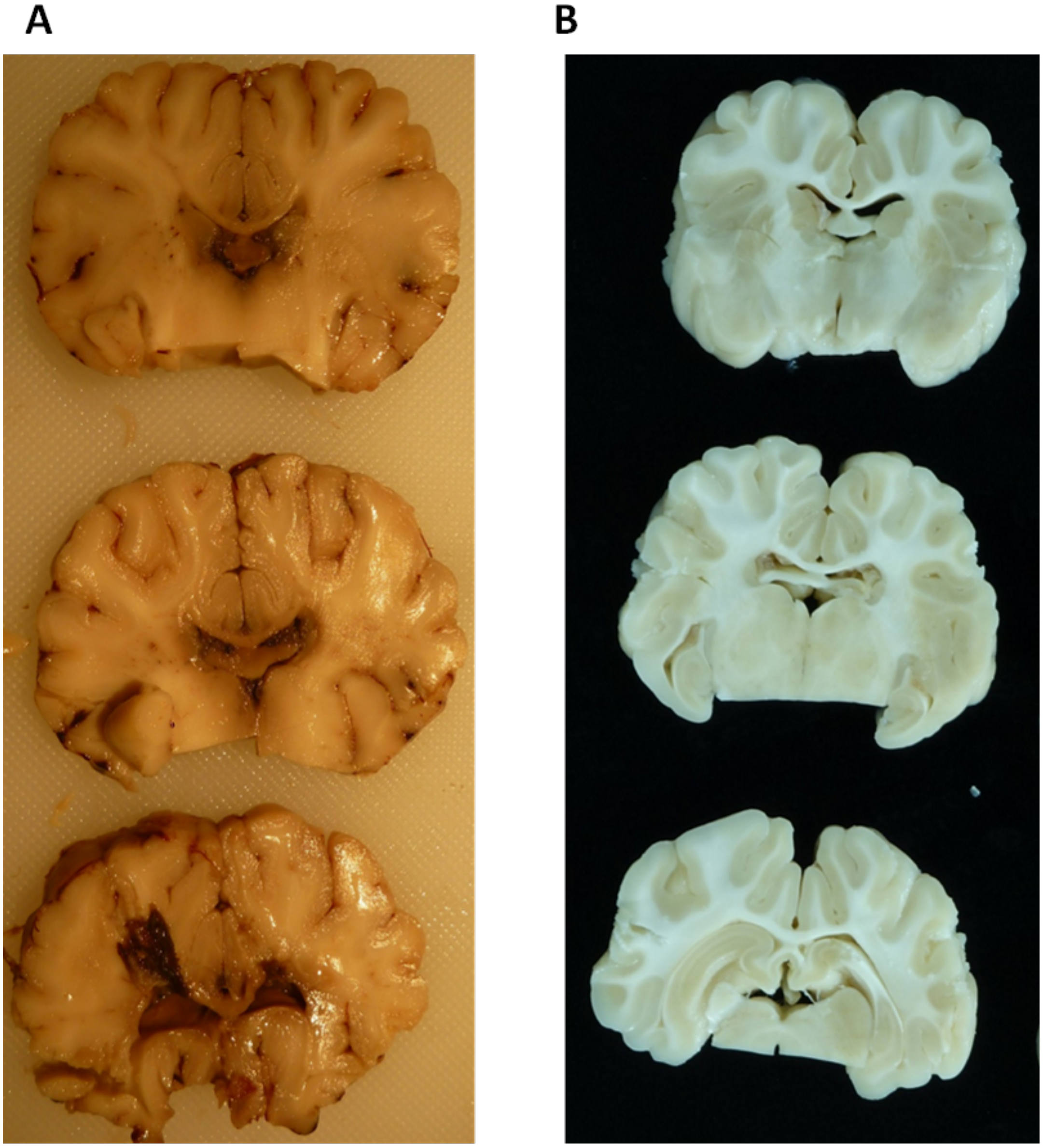

Supplementary Fig. 24 l Gross sections of brain tissue of swine on day 4 (enlarged photos). **A**: representative photos from control group revealed widespread injury in all areas, showing an overall dusky color, blurring of the gray white junction and intraventricular discoloration. **B**: representative photos from treatment group showed well preserved brain tissue without similar abnormalities.

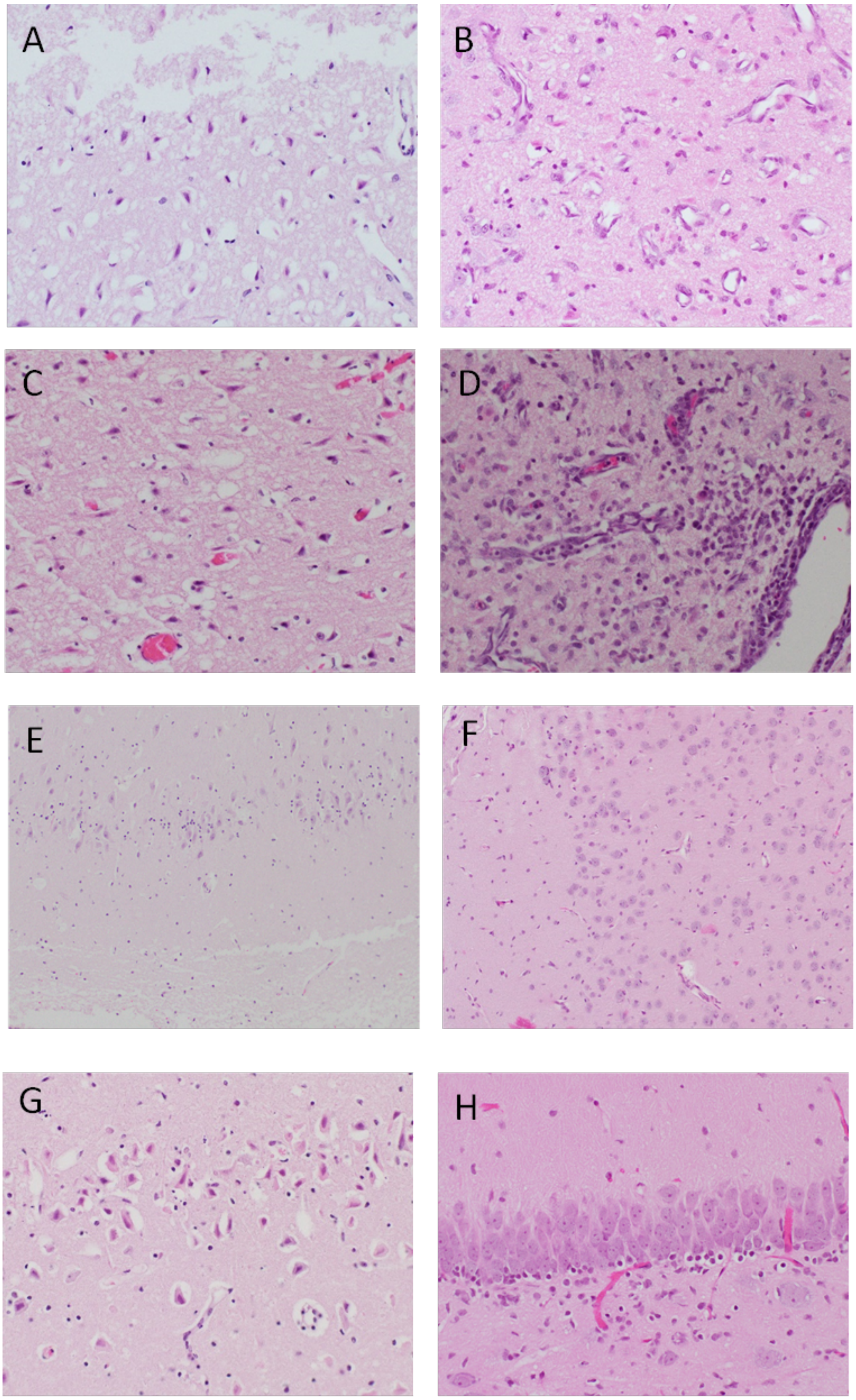
A
B
C
D
E
F
G
H

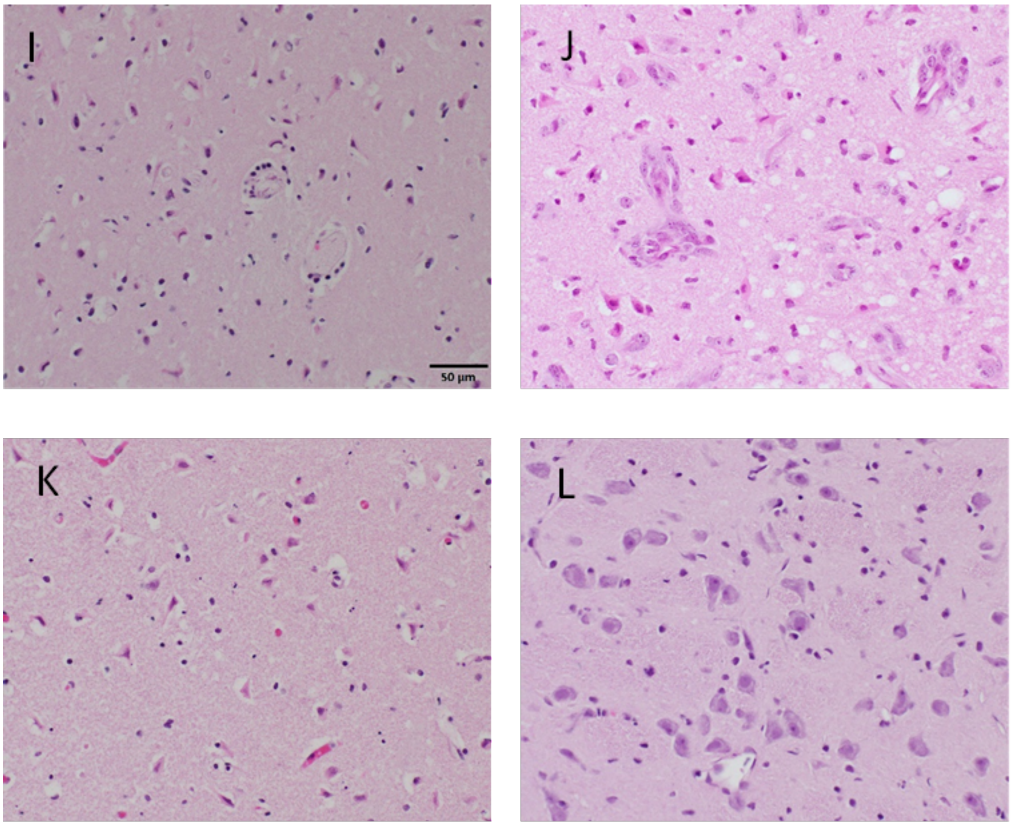

Supplementary Fig. 25 l Representative H&E-stained images taken at 20x or 40x magnification of isolated damage areas in different brain locations from swine study, with a severity score assigned by a pathologist blinded to treatment status. Frontal cortex: **A**, control, 4+; **B**, IVO2, 4 +. Parietal cortex: **C**, control, 4+; **D**, IVO2, 4+. Temporal cortex: **E**, control, 4+; **F**, IVO2, 0. Hippocampus: **G**, control, 4+; **H**, IVO2, 1+. Caudate: **I**, control, 4+; **J**, IVO2, 4+. Thalamus: **K**, control, 4+; **L**, IVO2, 0. Areas scored 4+ showed hyper eosinophilic neurons, apoptotic neuronal nuclei, vacuolated edematous neuropil (e.g., **A**, **C**) and reactive capillaries (e.g., **J**, **D**), whereas **F**, and **L** had no apparent injury.

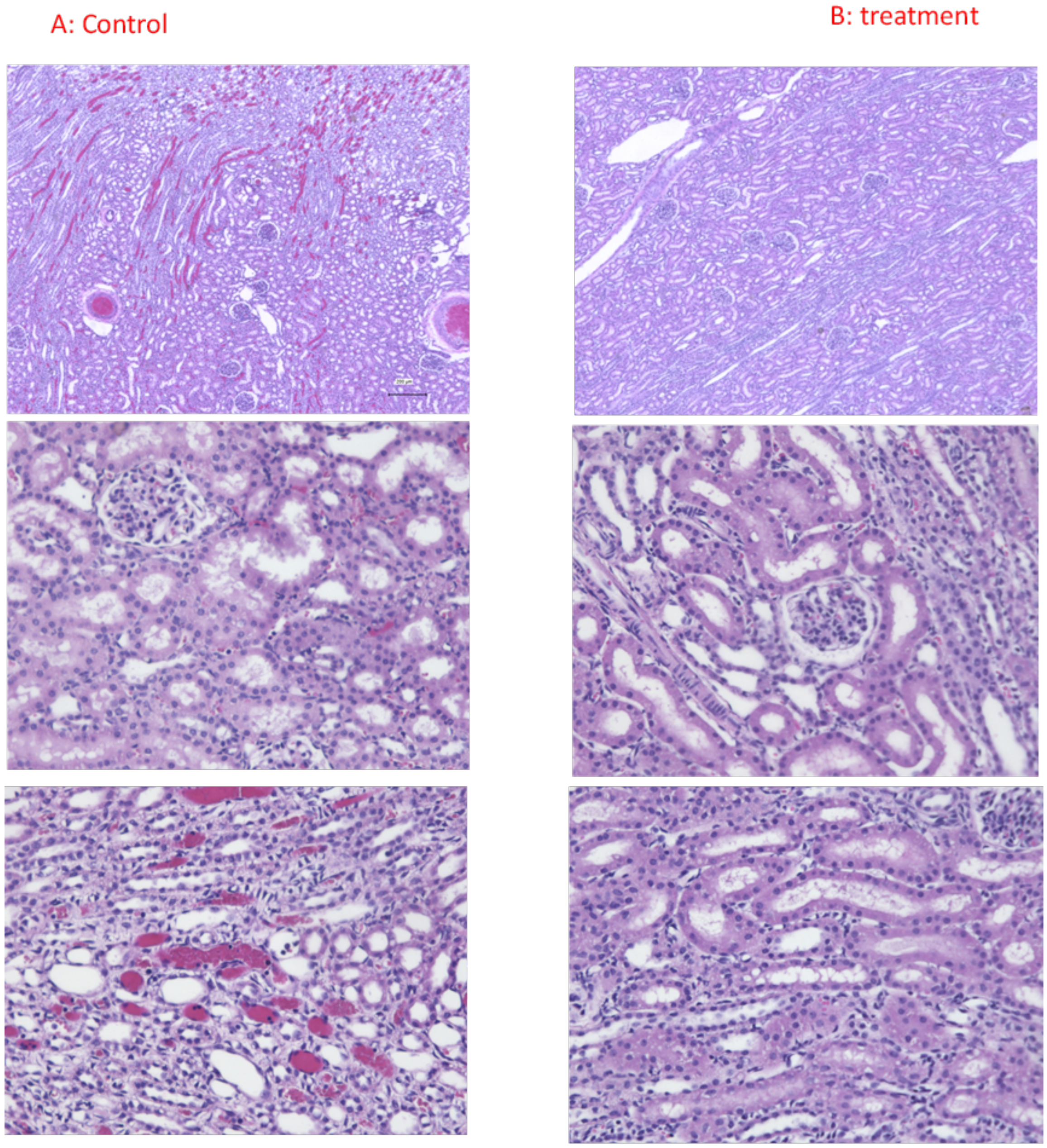

Supplementary Fig. 26 l Representative images of renal histology in swine asphyxia efficacy study. The control group (**A**, left side) showed typical signs of acute kidney injury including congested capillaries, tubular dilation and loss of brush borders, tubular degeneration as well as necrosis, where the treated group (**B**, right side) appeared to be normal.

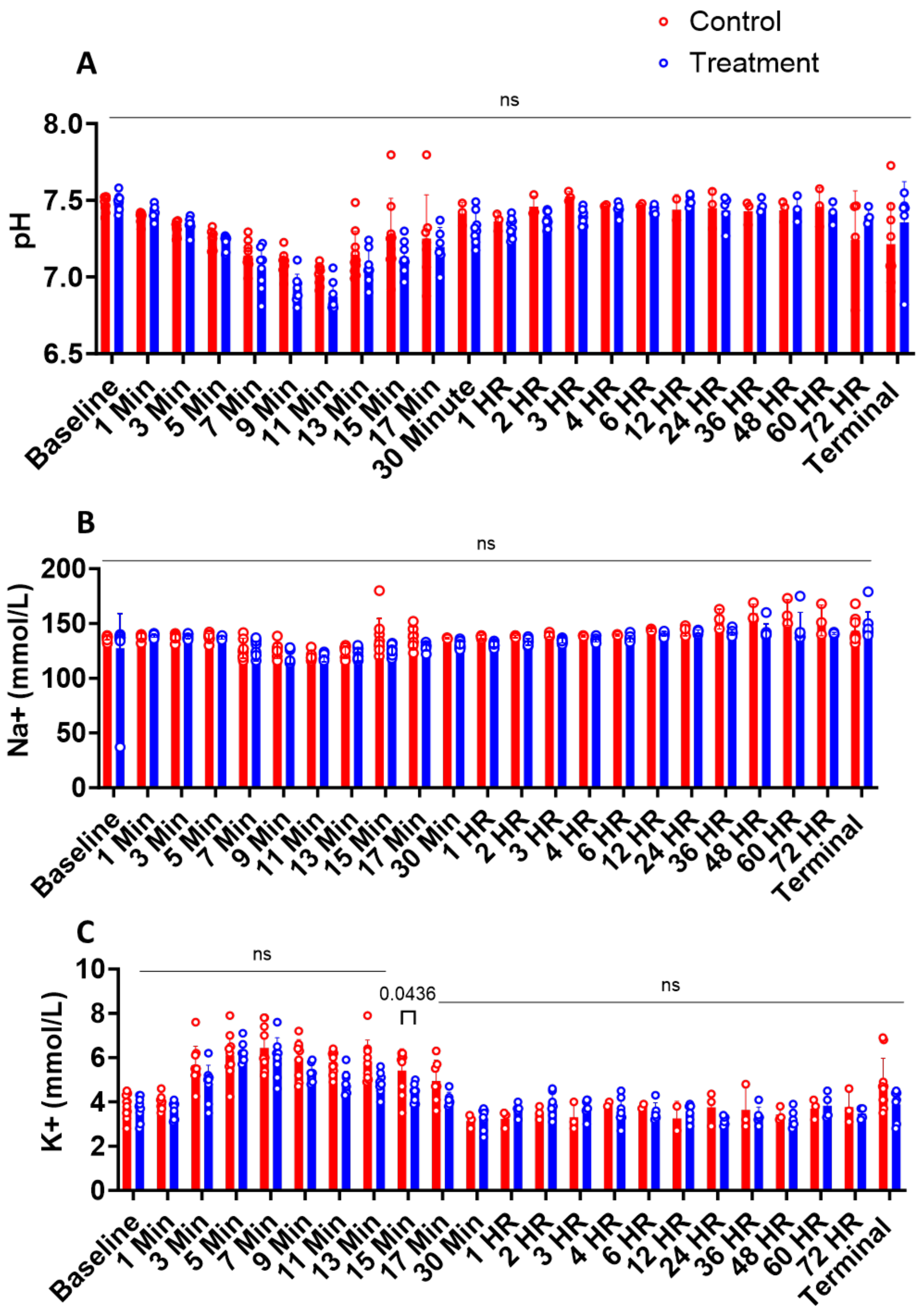
Control
Treatment
A
ns
pH
8.0
7.5
7.0
6.5
Baseline
1 Min
3 Min
5 Min
7 Min
9 Min
11 Min
13 Min
15 Min
17 Min
30 Minute
1 HR
2 HR
3 HR
4 HR
6 HR
12 HR
24 HR
36 HR
48 HR
60 HR
72 HR
Terminal
B
ns
Na+ (mmol/L)
200
150
100
50
0
Baseline
1 Min
3 Min
5 Min
7 Min
9 Min
11 Min
13 Min
15 Min
17 Min
30 Min
1 HR
2 HR
3 HR
4 HR
6 HR
12 HR
24 HR
36 HR
48 HR
60 HR
72 HR
Terminal
C
ns
0.0436
ns
K+ (mmol/L)
10
8
6
4
2
0
Baseline
1 Min
3 Min
5 Min
7 Min
9 Min
11 Min
13 Min
15 Min
17 Min
30 Min
1 HR
2 HR
3 HR
4 HR
6 HR
12 HR
24 HR
36 HR
48 HR
60 HR
72 HR
Terminal

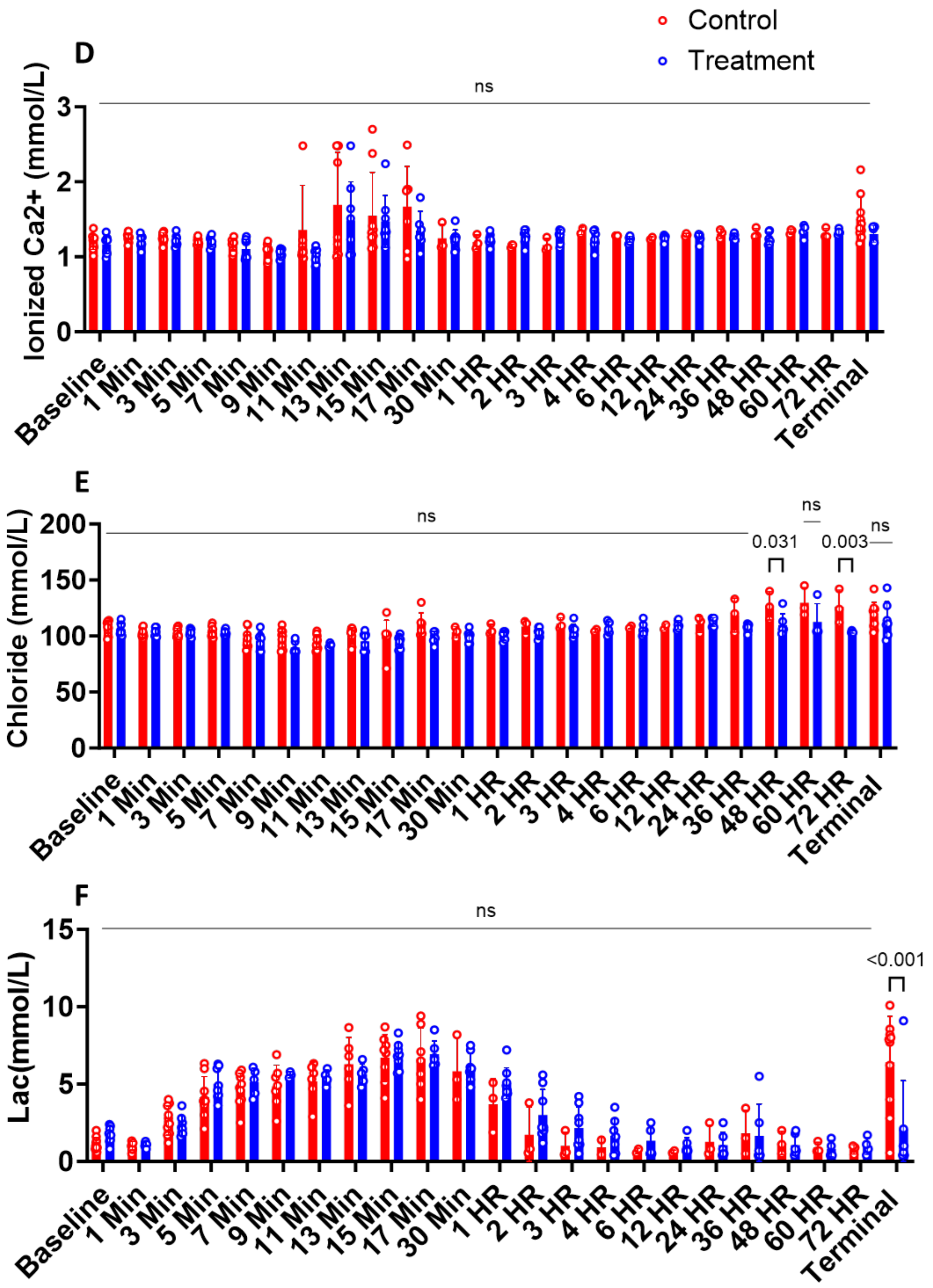

Supplementary Fig. 27 l Changes in serum electrolytes in asphyxia efficacy study. **A**, pH. **B**, sodium. C, potassium. **D**, ionized calcium. **E**, chloride. **F**, lactate. Data presented as mean ± SD, statistical analysis by two-way ANOVA between control and treatment groups. Infusion of PMBs did not lead to significant decrease in blood pH and did not show any abnormalities in all markers.

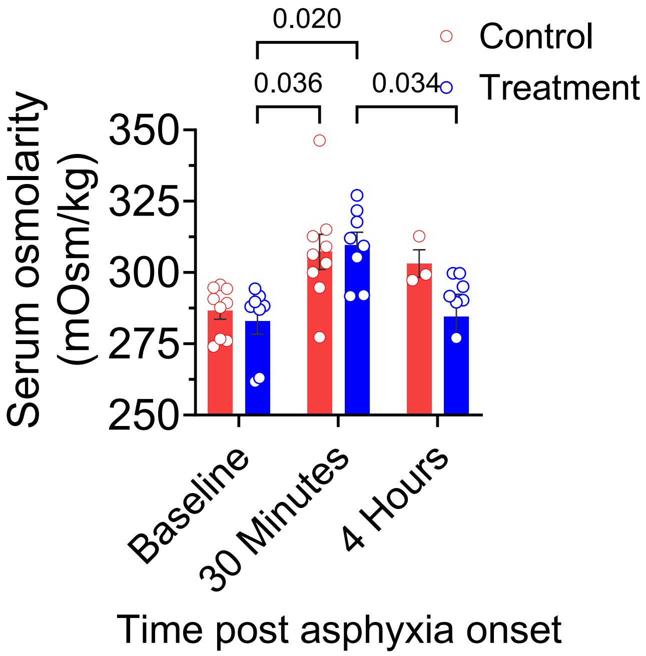

Supplementary Fig. 28 I Serum osmolarity increased compared with baseline at 30 minutes equally in control and IVO2-treated groups; this is expected since a significant volume of hypertonic D10 was infused in both groups. At 4-hour post asphyxia, the blood osmolarity returned to normal in both groups. Data presented as mean ± SD. Statistical analysis by a two-way ANOVA test.

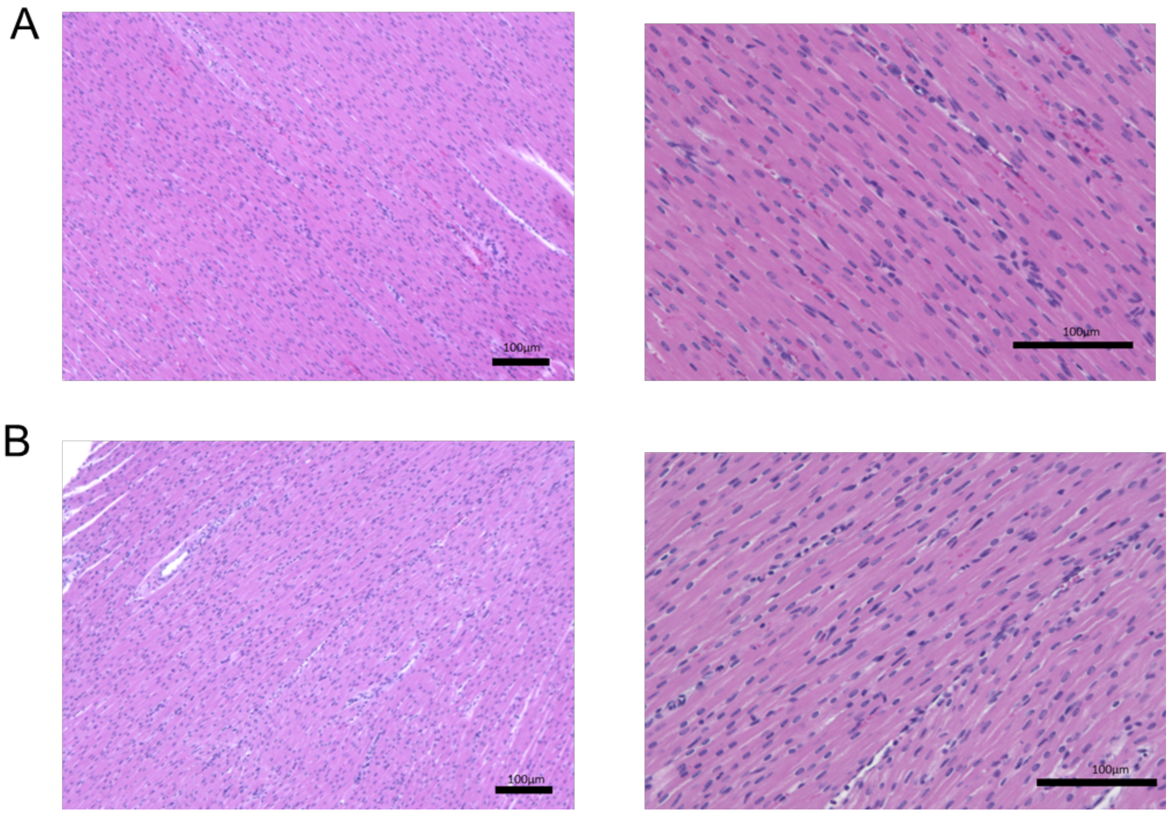

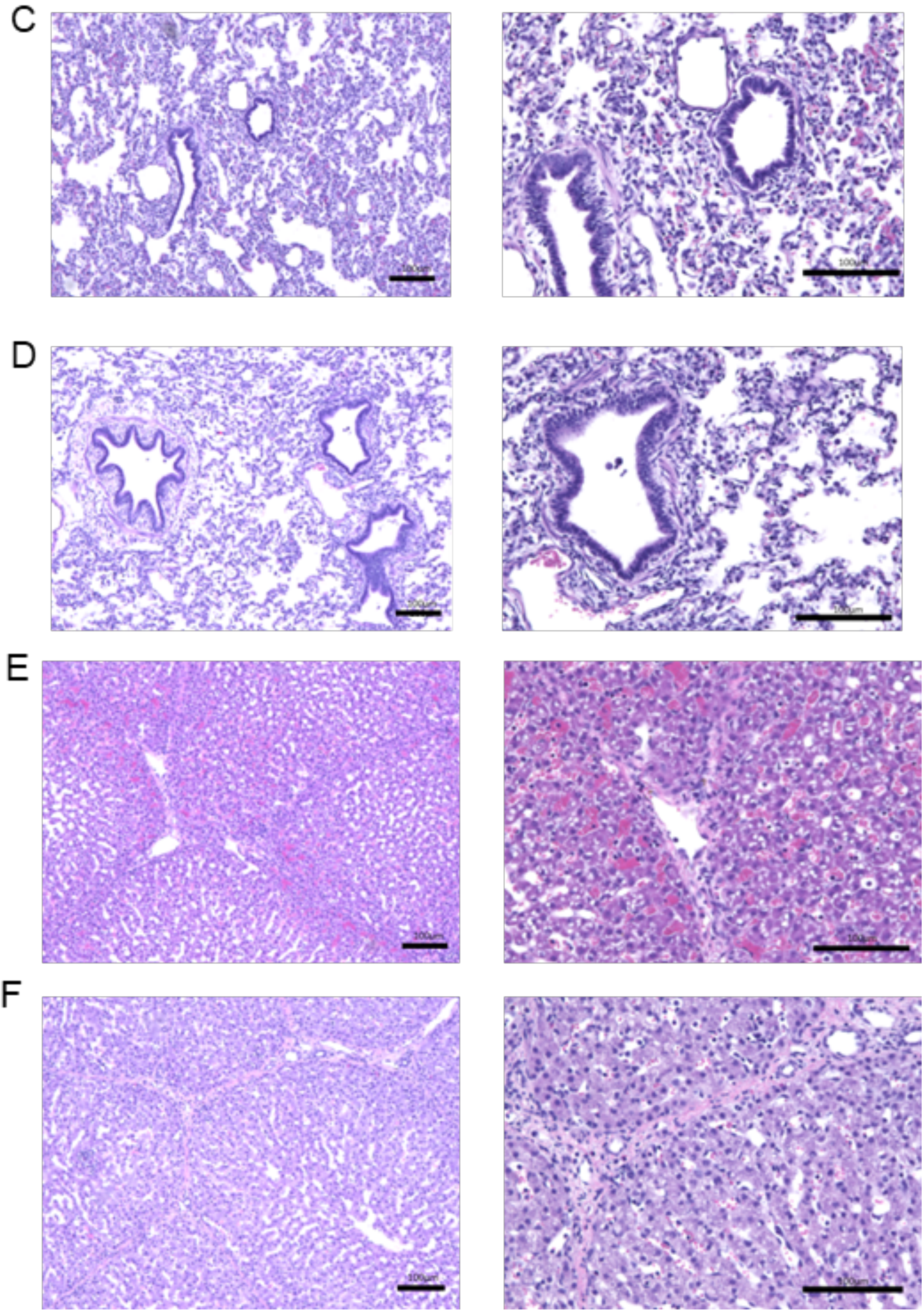
C
D
E
F

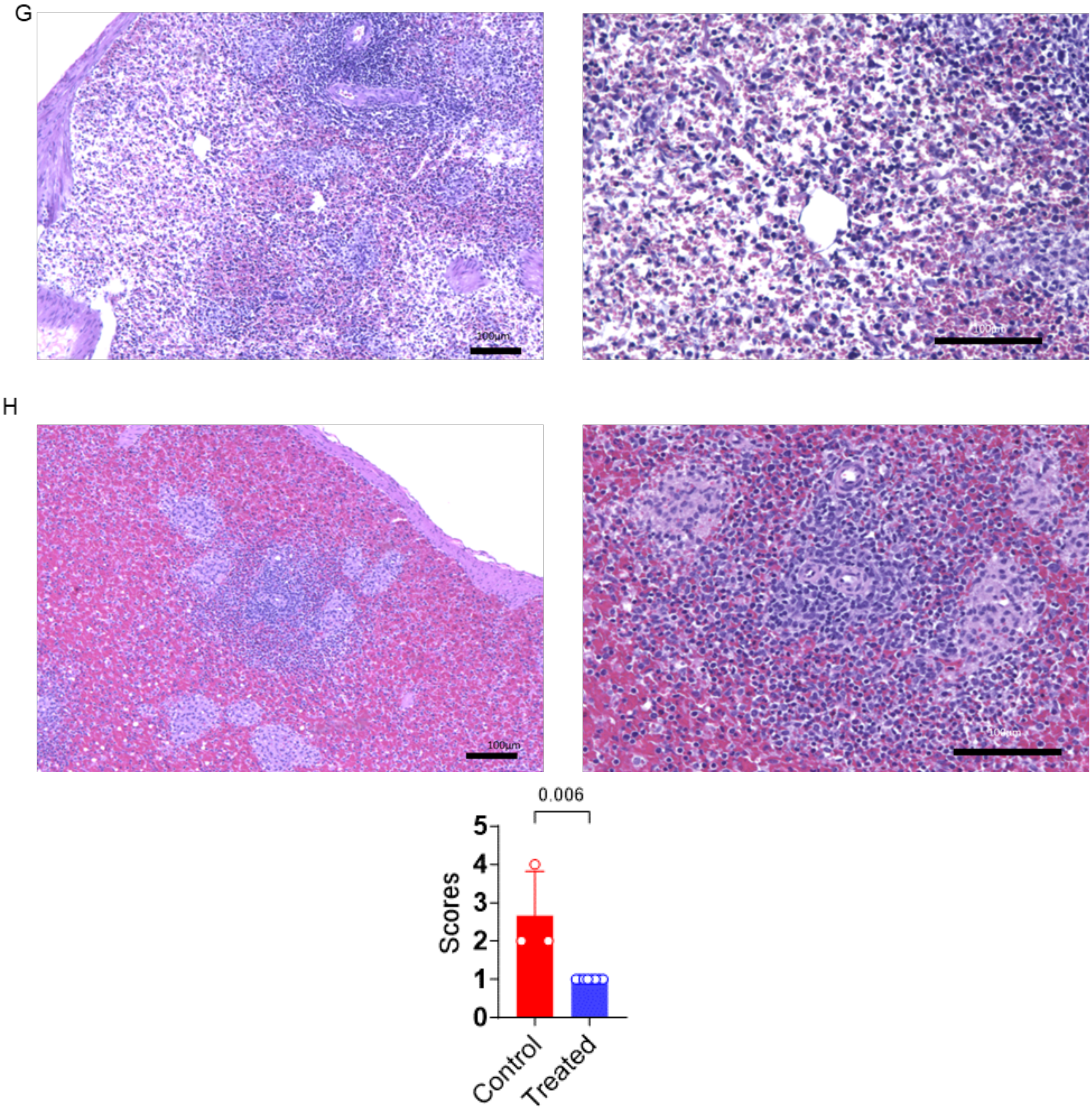

Supplementary Fig. 29 l Representative H&E-stained histological images of vital organs of survived swine in treatment and control groups. Heart: control (**A**) and treatment (**B**). Lung: control (**C**) and treatment (**D**). Liver: control (**E**) and treatment (**F**). Spleen: control (**G**) and treatment (**H**). Both groups appear to show normal histology in heart and lung. The livers in control group (**E**) showed signs of congested blood vessels, while those in treatment group appeared to be normal. The spleen in control group (**G**) showed little to no hemopoiesis in the red pulp, likely indicating further injury due to severe hypoxia; where the spleen of treated animals (**H**) appeared to be normal. The hematopoiesis was blindly scored by a pathologist as 0-1: normal, 1-2, mild, 2-3 severe, 3-4 extreme (Data presented as mean, error = SD, Mann-Whitney test).

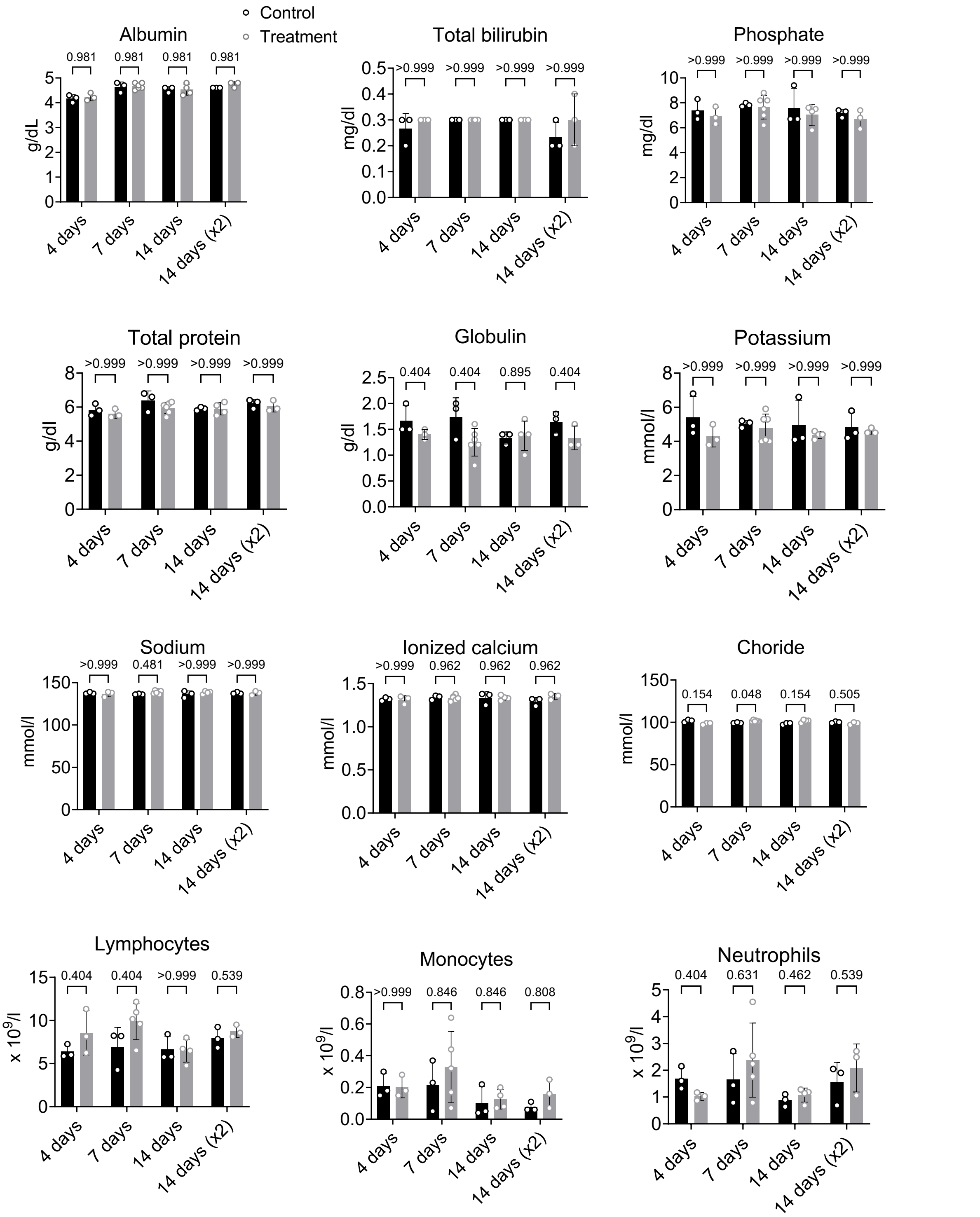

Supplementary Fig. 30 I Changes in clinical markers in rodent safety study. Data presented as mean ± SD, and the statistical analysis by Mann-Whitney test with p values shown in the plots. Treated animals receiving both dosages showed no significant difference from control groups receiving equal volume of D10, all values were within normal ranges.

A
1

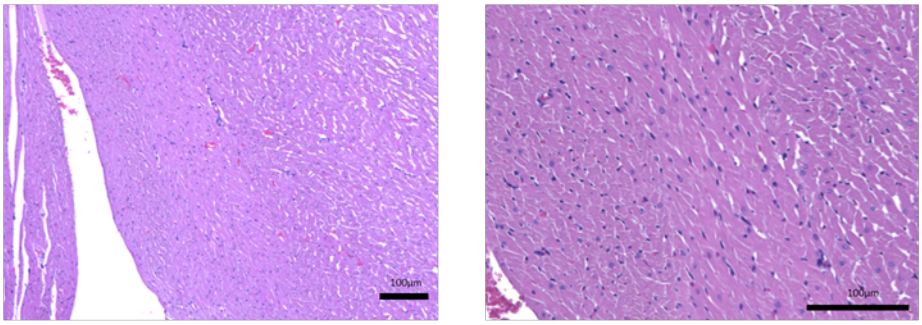

A
2

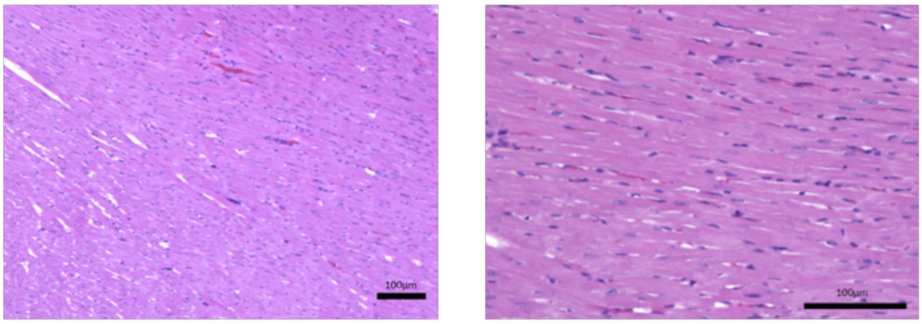

B
1

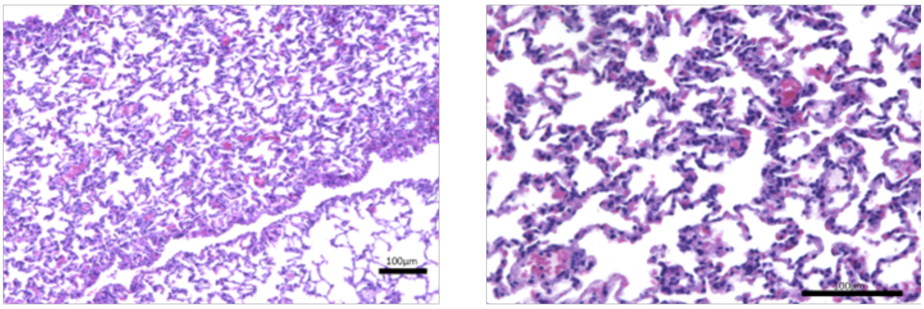

B
2

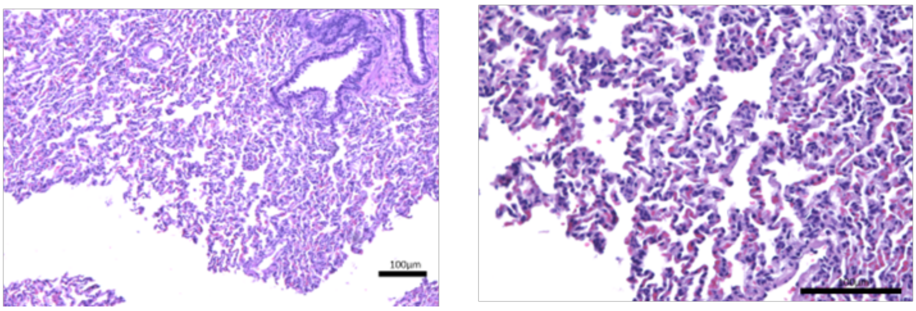

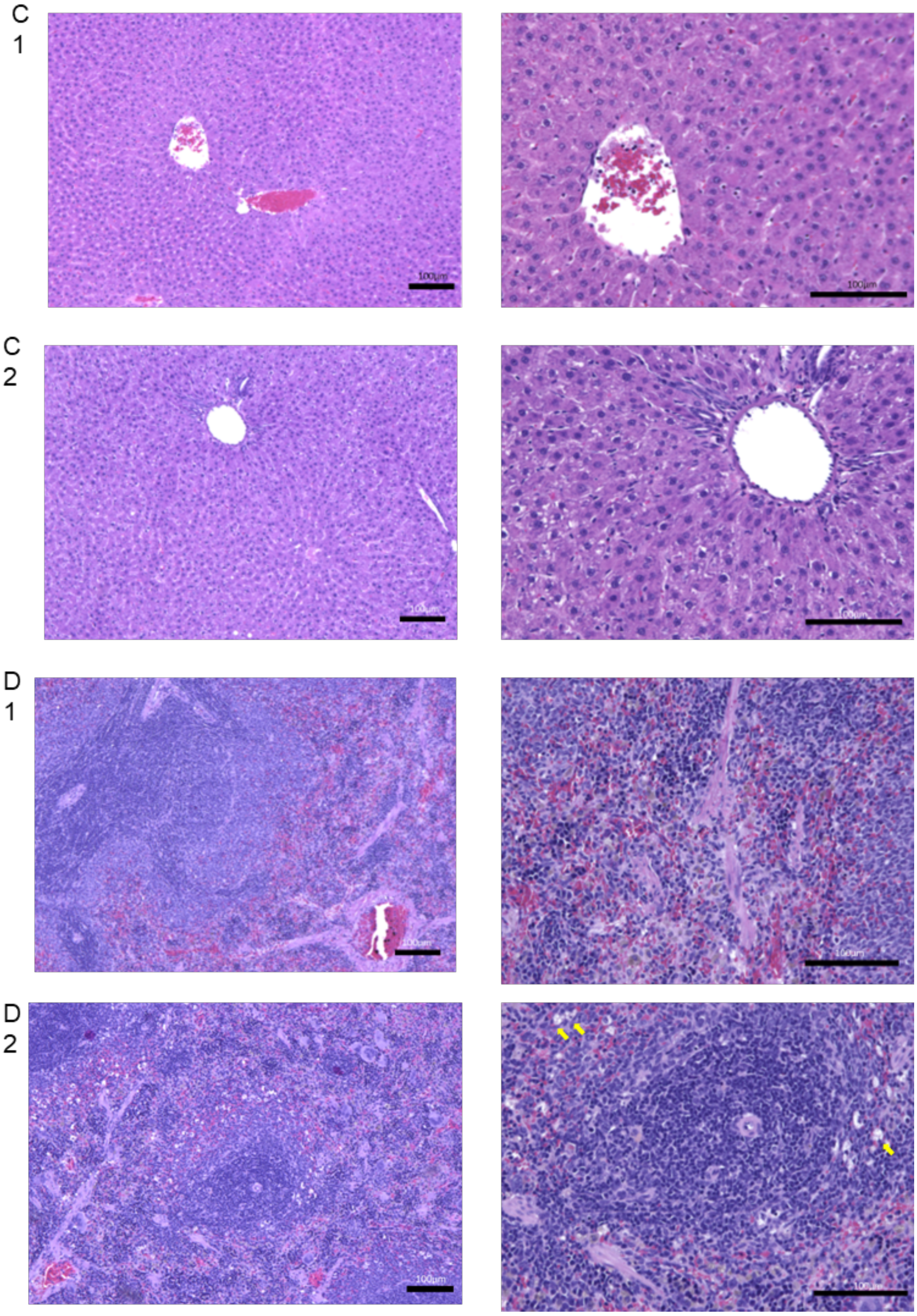
C
1
C
2
D
1
D
2

E 1

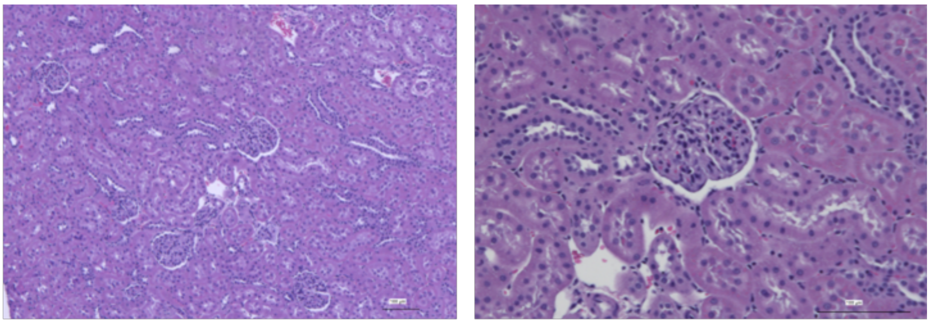

E 2

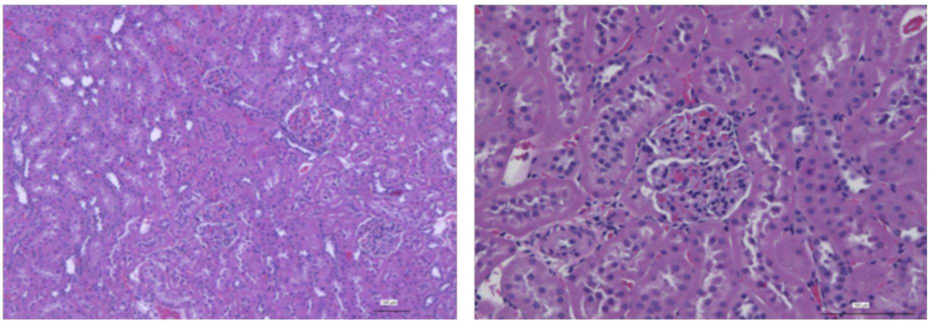

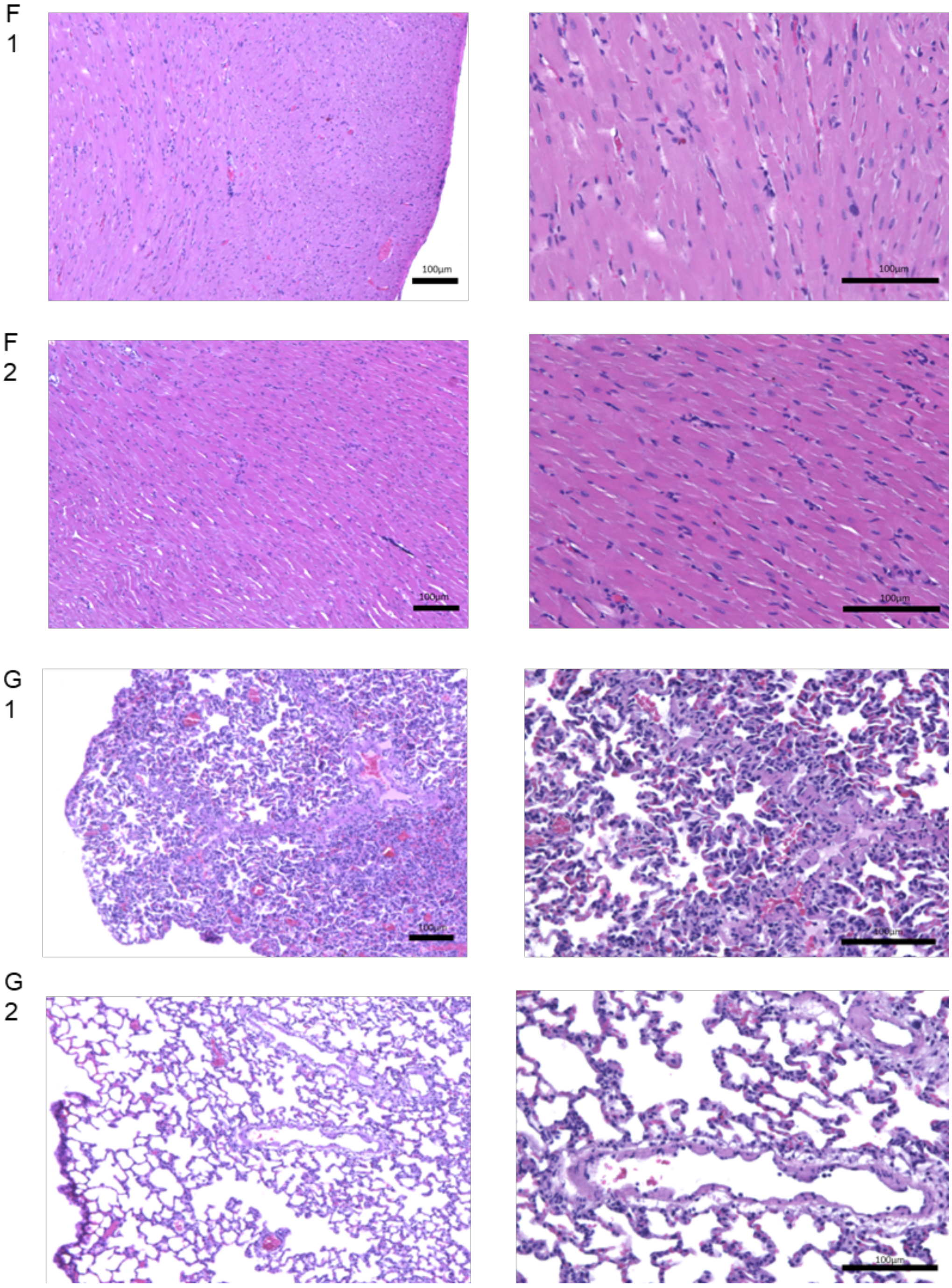

F 1
100μm
100μm
F 2
100μm
100μm
G 1
100μm
G 2
100μm

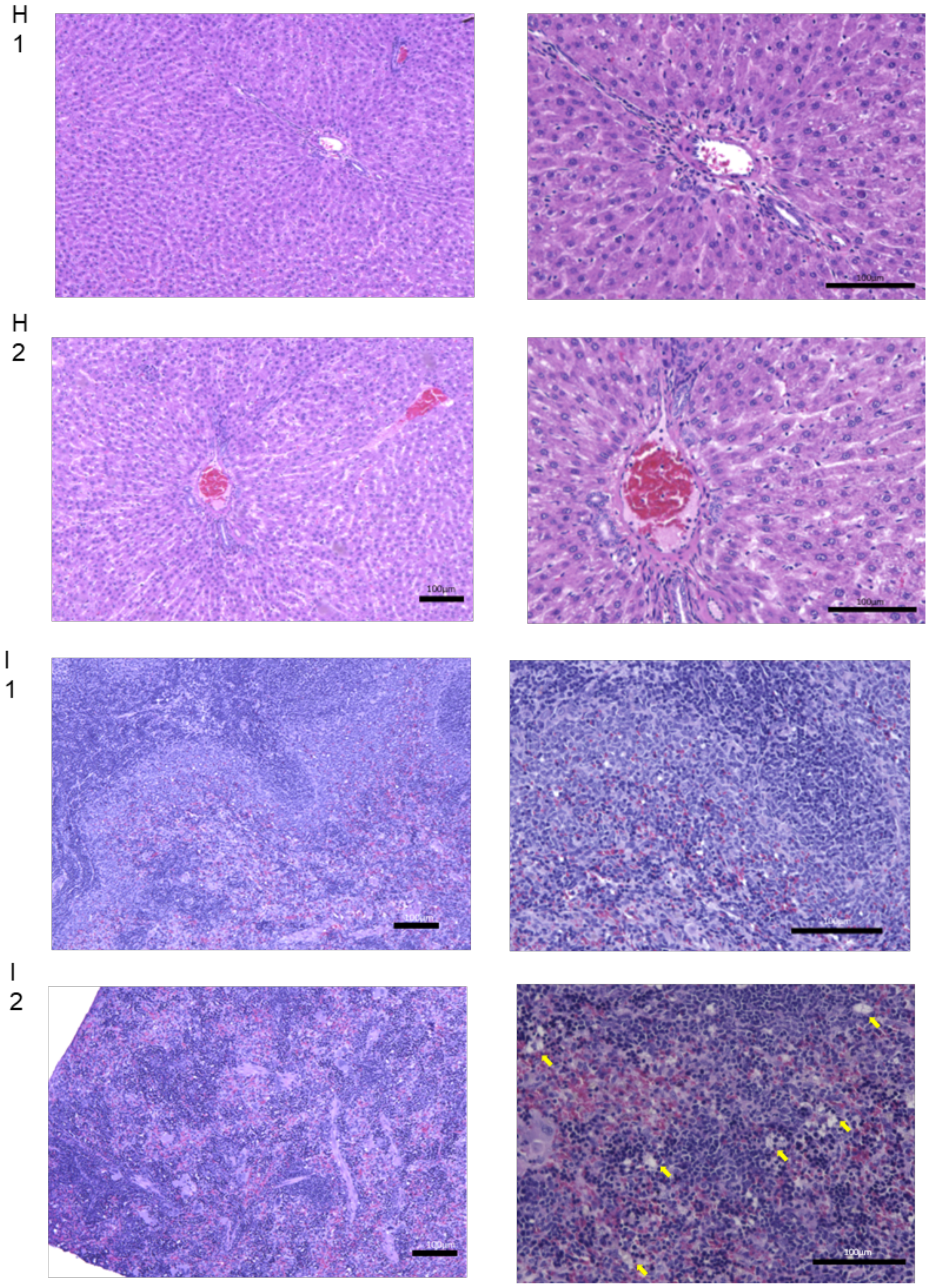
H
1
100μm
H
2
100μm
100μm
I
1
100μm
I
2
100μm
100μm

J
1

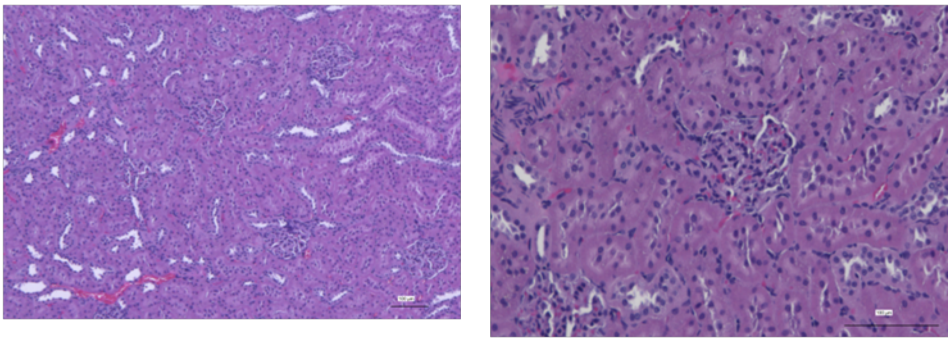

J
2

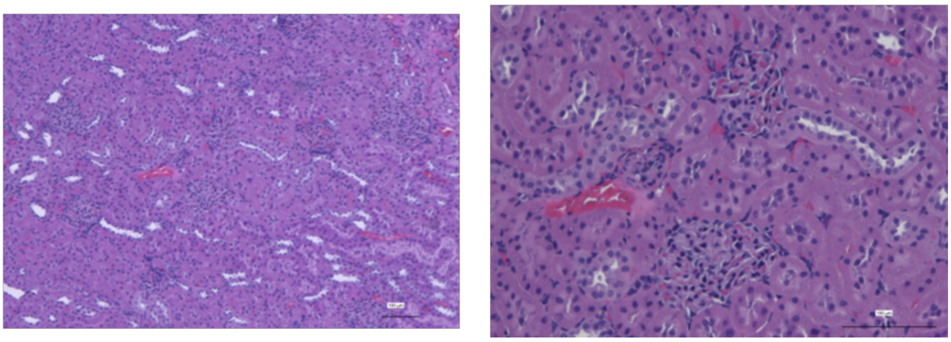

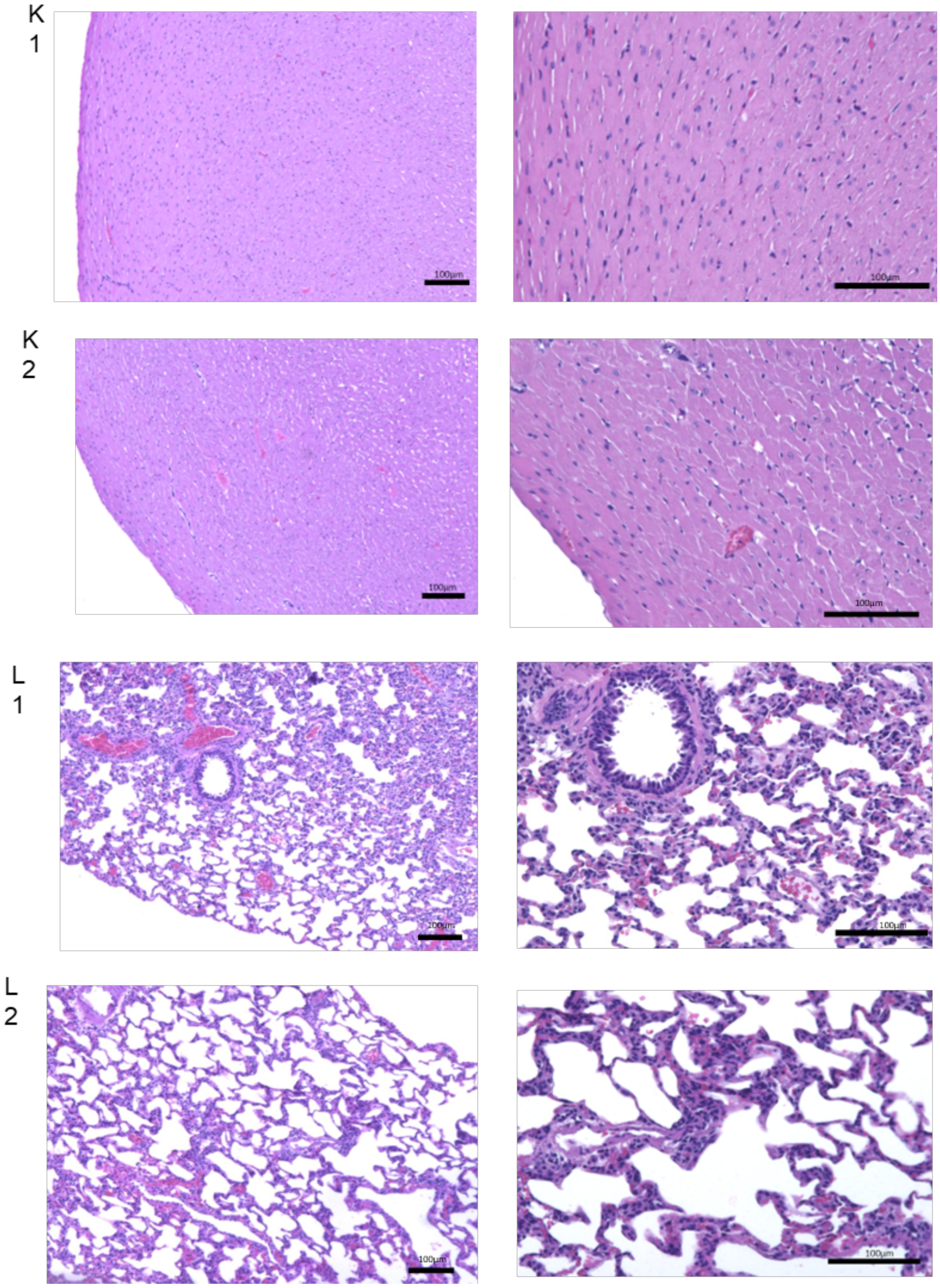
K
1
100μm
100μm
K
2
100μm
100μm
L
1
100μm
100μm
L
2
100μm
100μm

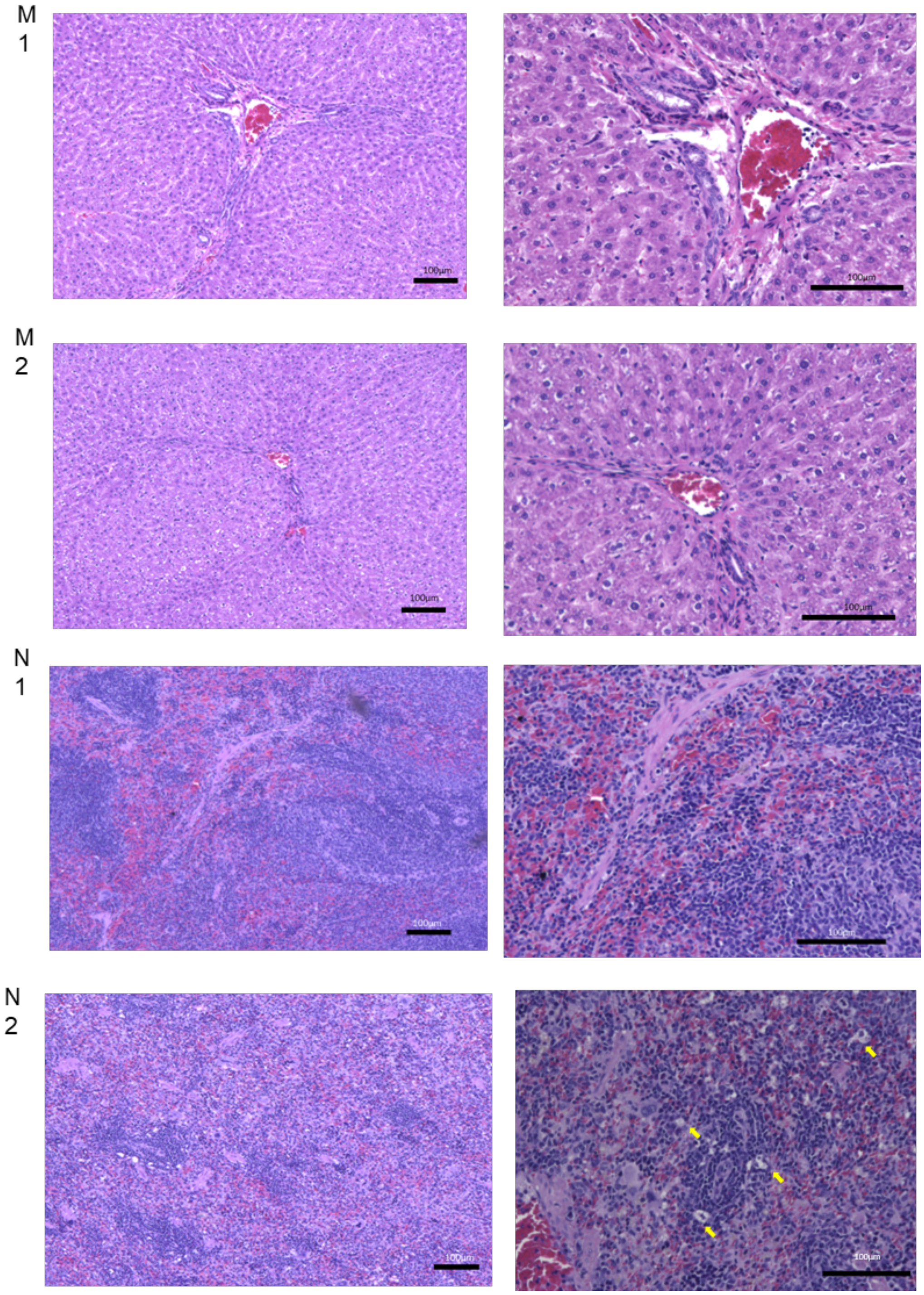
M
1
M
2
N
1
N
2
100μm

O
1

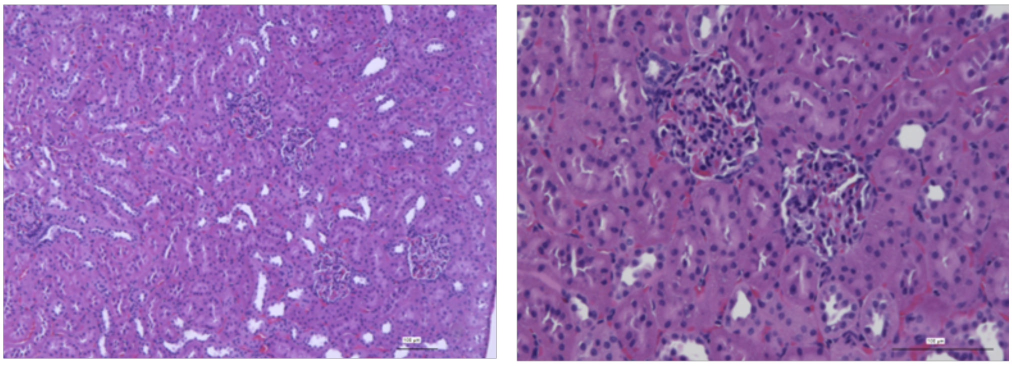

O
2

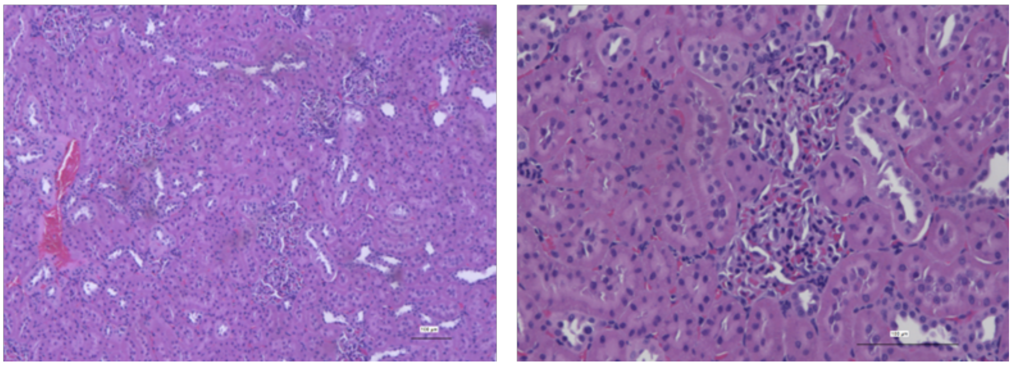

P 1

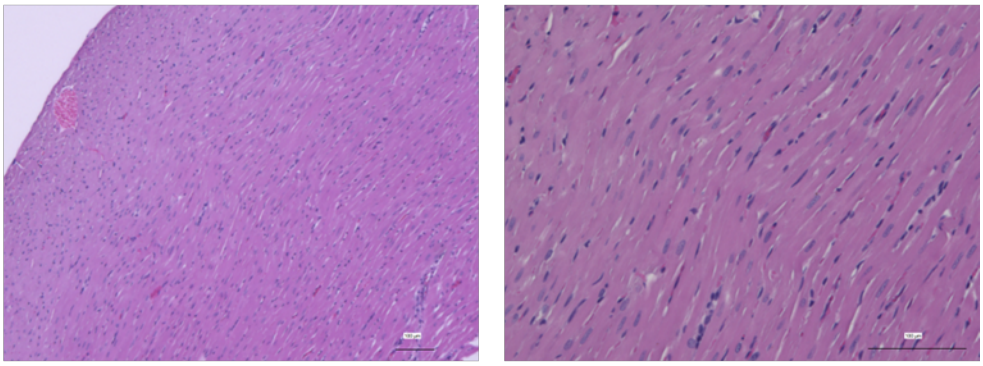

P 2

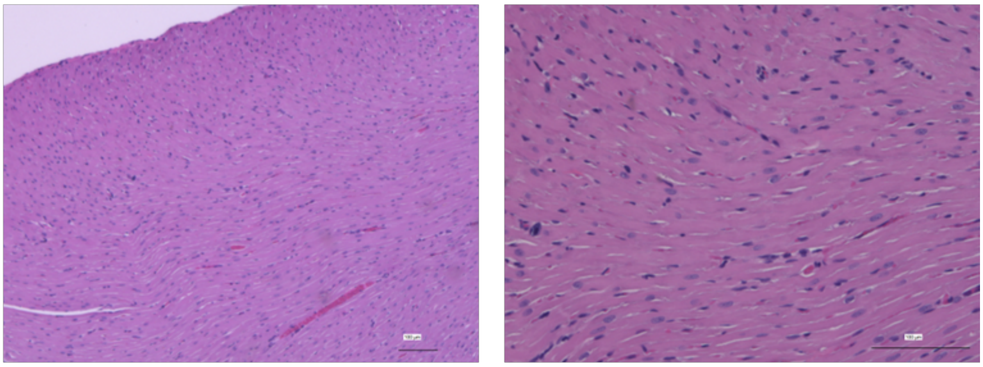

Q 1

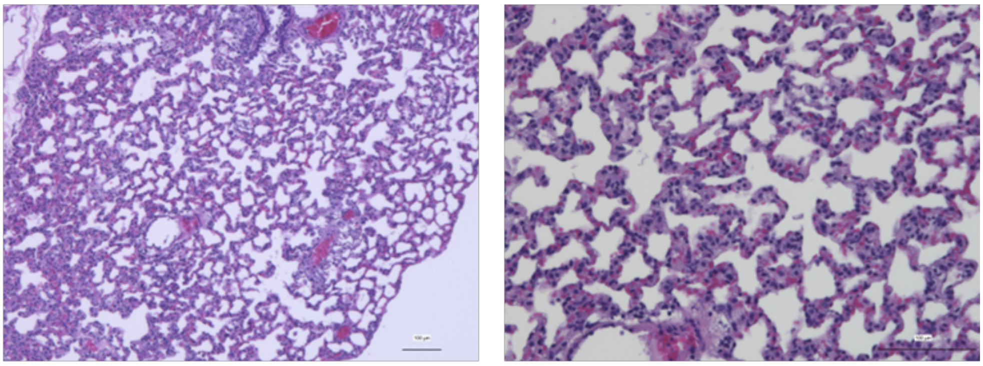

Q 2

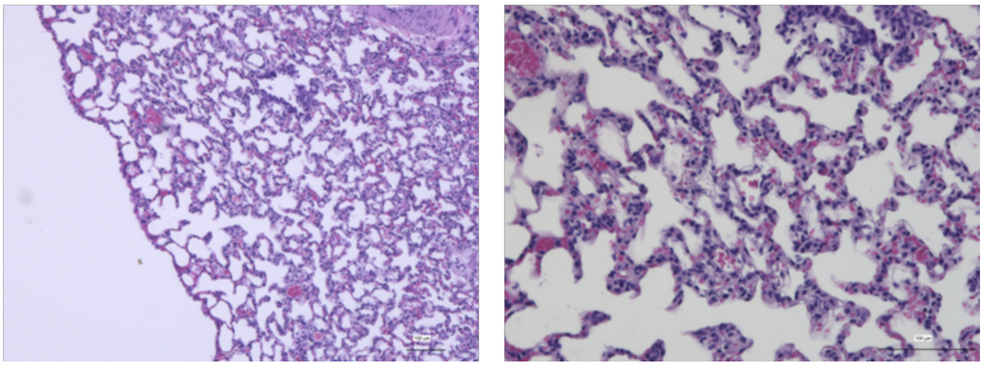

R
1

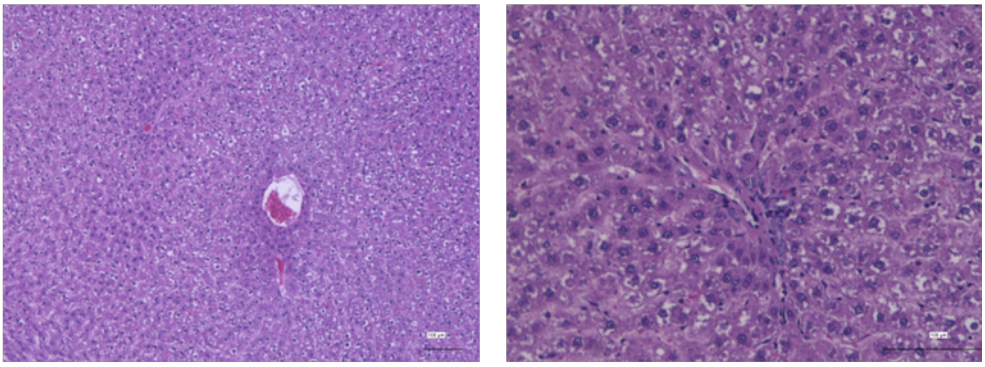

R
2

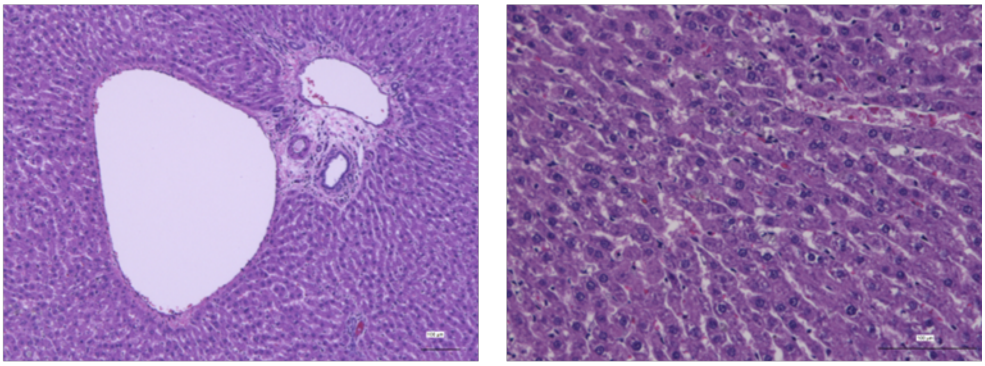

S
1

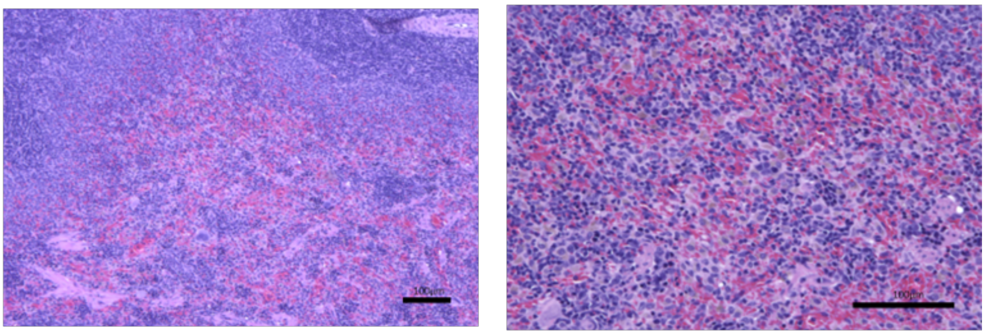

S
2

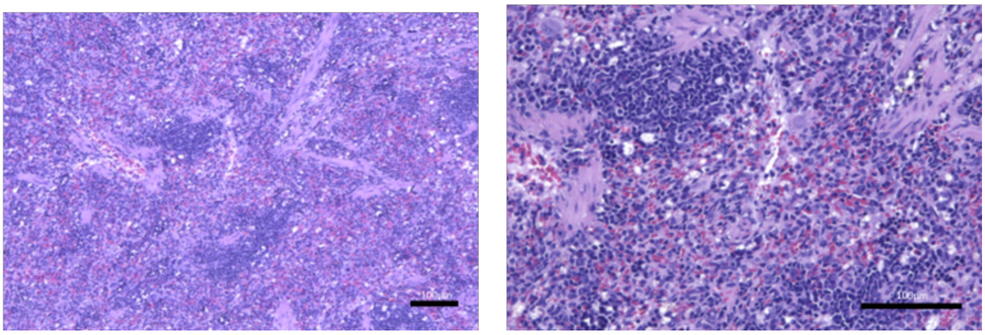

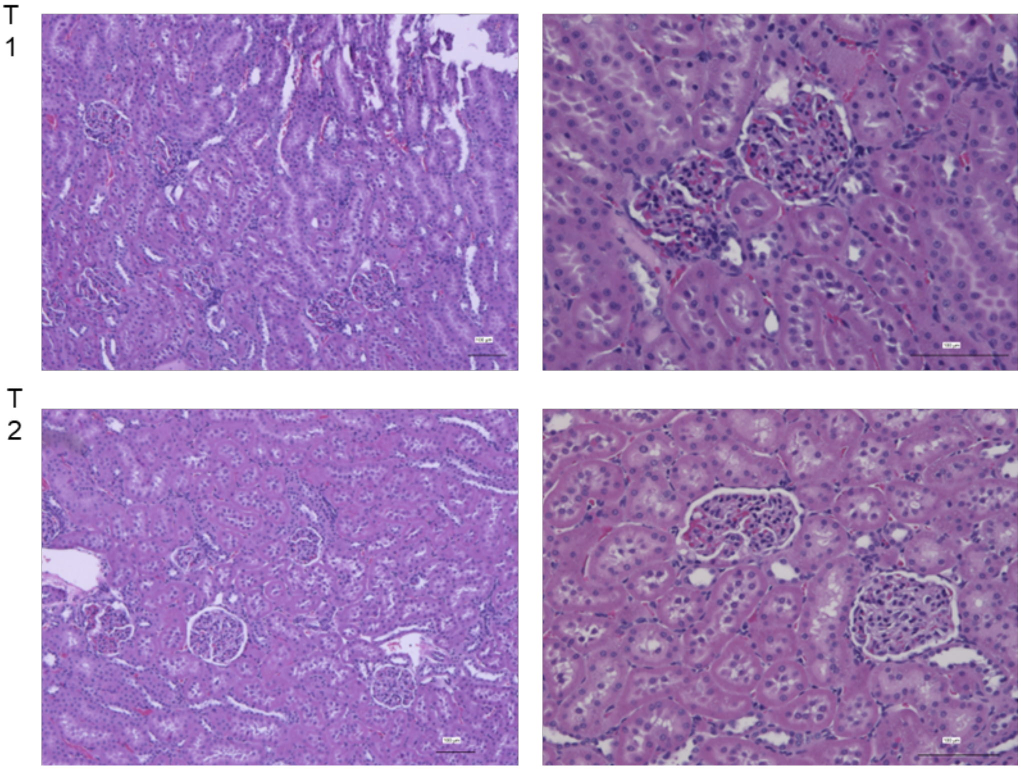

Supplementary Fig. 31 l Representative histological images in rodent safety study. **1**: control group; **2** treatment group. [Dosage 1] 4 days: **A**, heart; **B**, lung; **C**, liver; **D** spleen; **E**, kidney. 7 days: **F**, heart; **G**, lung; **H**, liver; **I**, spleen; **J**, kidney. 14 days: **K**, heart; **L**, lung; **M**, liver; **N** spleen; **O**, kidney. [Dosage 2] 14 days: **P**, heart; **Q**, lung; **R**, liver; **S**, spleen; **T**, kidney. At all-time points, no histological abnormalities were seen in the heart, lung, liver, or kidney. In the treatment group splenic macrophage vacuole formation was observed.

Supplementary Video 1
Rapid dissolution of air-filled PMBs when added into air-saturated PBS buffer at pH 7.2. Disappearance of white colors indicates bubble dissolutions.

Supplementary Video 2
Echocardiography of a rat heart (no injection as control).

Supplementary Video 3
Echocardiography of a rat heart during infusion of lipid oxygen microbubbles (LOMs) at 4 ml gas/kg/minute.

Supplementary Video 4
Echocardiography of a rat heart during infusion of oxygen-filled oPMBs at 4 ml gas/kg/minute.

Supplementary Video 5
Echocardiography of a rat heart during infusion of air-filled aPMBs at 4 ml gas/kg/minute.
