# Supplementary material for: Systemically injected oxygen within rapidly dissolving microbubbles improves the outcomes of severe hypoxaemia in swine
Source: Nat Biomed Eng. 2024 Oct 17;8(11):1396–411. doi: 10.1038/s41551-024-01266-8 (PMC11584390; doi:10.1038/s41551-024-01266-8)
Supplement: Supplementary file 2 — Reporting Summary [file 41551_2024_1266_MOESM2_ESM.pdf]

nature portfolio

Corresponding author(s): John Kheir and Yifeng Peng

Last updated by author(s): May 20, 2024

Reporting Summary

Nature Portfolio wishes to improve the reproducibility of the work that we publish. This form provides structure for consistency and transparency in reporting. For further information on Nature Portfolio policies, see our Editorial Policies and the Editorial Policy Checklist.

Statistics

For all statistical analyses, confirm that the following items are present in the figure legend, table legend, main text, or Methods section.

n/a	Confirmed	
☐	☒	The exact sample size (*n*) for each experimental group/condition, given as a discrete number and unit of measurement
☐	☒	A statement on whether measurements were taken from distinct samples or whether the same sample was measured repeatedly
☐	☒	The statistical test(s) used AND whether they are one- or two-sided<br>*Only common tests should be described solely by name; describe more complex techniques in the Methods section.*
☐	☒	A description of all covariates tested
☐	☒	A description of any assumptions or corrections, such as tests of normality and adjustment for multiple comparisons
☐	☒	A full description of the statistical parameters including central tendency (e.g. means) or other basic estimates (e.g. regression coefficient) AND variation (e.g. standard deviation) or associated estimates of uncertainty (e.g. confidence intervals)
☐	☒	For null hypothesis testing, the test statistic (e.g. *F*, *t*, *r*) with confidence intervals, effect sizes, degrees of freedom and *P* value noted<br>*Give P values as exact values whenever suitable.*
☒	☐	For Bayesian analysis, information on the choice of priors and Markov chain Monte Carlo settings
☒	☐	For hierarchical and complex designs, identification of the appropriate level for tests and full reporting of outcomes
☒	☐	Estimates of effect sizes (e.g. Cohen's *d*, Pearson's *r*), indicating how they were calculated

Our web collection on statistics for biologists contains articles on many of the points above.

Software and code

Policy information about availability of computer code

Data collection	Bruker Topspin was used to collected NMR data. No software was used in other data collections.
Data analysis	All data was analysed using GraphPad Prism version 10.1.2 for Windows GraphPad Software. MRI results were analysed by an open-source ITK-SNP 4.0 software application (http://www.itksnap.org/pmwiki/pmwiki.php; Penn Image Computing and Science Laboratory, University of Pennsylvania, Pennsylvania, and Scientific Computing and Imaging Institute, University of Utah, Utah).

For manuscripts utilizing custom algorithms or software that are central to the research but not yet described in published literature, software must be made available to editors and reviewers. We strongly encourage code deposition in a community repository (e.g. GitHub). See the Nature Portfolio guidelines for submitting code & software for further information.

Data

Policy information about availability of data

All manuscripts must include a data availability statement. This statement should provide the following information, where applicable:

- Accession codes, unique identifiers, or web links for publicly available datasets
- A description of any restrictions on data availability
- For clinical datasets or third party data, please ensure that the statement adheres to our policy

The main data supporting the results in this study are available within the paper and its Supplementary Information. Source data are provided with this paper.

Additional microscopic and ultrasound images, experimental notes, and raw (animal) patient data generated during the study are available from the corresponding authors on reasonable request.

Research involving human participants, their data, or biological material

Policy information about studies with human participants or human data. See also policy information about sex, gender (identity/presentation), and sexual orientation and race, ethnicity and racism.

Reporting on sex and gender	The study did not involve human research participants.
Reporting on race, ethnicity, or other socially relevant groupings	–
Population characteristics	–
Recruitment	–
Ethics oversight	–

Note that full information on the approval of the study protocol must also be provided in the manuscript.

Field-specific reporting

Please select the one below that is the best fit for your research. If you are not sure, read the appropriate sections before making your selection.

☒ Life sciences ☐ Behavioural & social sciences ☐ Ecological, evolutionary & environmental sciences

For a reference copy of the document with all sections, see nature.com/documents/nr-reporting-summary-flat.pdf

Life sciences study design

All studies must disclose on these points even when the disclosure is negative.

Sample size	On the basis of preliminary data, we expected a Swine Neurologic Deficit Score of 420 with s.d. of 100 in surviving swine in the control group. The inclusion of 3 surviving animals per group allowed us to detect a difference of 250 in the SNDS at 3 days with 90% power and an alpha of 0.05.
Data exclusions	No data were excluded.
Replication	All instruments were calibrated prior to use.
Randomization	Swine were randomized between groups in alternating fashion.
Blinding	The investigators performing experiments were not blinded to the treatment group because the difference in haemodynamics was marked and obvious to all in the room. The investigators reading the MRI and histology endpoints were blinded to treatment allocation.

Reporting for specific materials, systems and methods

We require information from authors about some types of materials, experimental systems and methods used in many studies. Here, indicate whether each material, system or method listed is relevant to your study. If you are not sure if a list item applies to your research, read the appropriate section before selecting a response.

Materials & experimental systems

n/a	Involved in the study	
☒	☐	Antibodies
☒	☐	Eukaryotic cell lines
☒	☐	Palaeontology and archaeology
☐	☒	Animals and other organisms
☒	☐	Clinical data
☒	☐	Dual use research of concern
☒	☐	Plants

Methods

n/a	Involved in the study	
☒	☐	ChIP-seq
☒	☐	Flow cytometry
☐	☒	MRI-based neuroimaging

April 2023

Animals and other research organisms

Policy information about studies involving animals; ARRIVE guidelines recommended for reporting animal research, and Sex and Gender in Research

Laboratory animals	Female Yorkshire swine, <1 month; Male Sprage Dawley rats, ~500 grams.
Wild animals	The study did not involve wild animals.
Reporting on sex	Single sex was used for each study, as describe above.
Field-collected samples	The study did not involve samples collected from the field.
Ethics oversight	Boston Children's Hospital Institutional Animal Care and Use Committee.

Note that full information on the approval of the study protocol must also be provided in the manuscript.

Magnetic resonance imaging

Experimental design

Design type	MRI performed at a single time point at the end of the swine experiments.
Design specifications	One time imaging for each subject at 84 hours post-injury.
Behavioral performance measures	The swine neurologic deficit score was compared between groups using 2-way ANOVA.

Acquisition

Imaging type(s)	Structural MRI, DWI, MRA.
Field strength	3T
Sequence & imaging parameters	T1-MPRAGE: TE 2.5ms, TI 900ms, TR 1600ms, Matrix size 184x256, FoV 140mmx196mm, FA 9°, THK 0.9mm.<br>T2-weighted TSE:TE 100ms, TR 10,400ms, Matrix size 291x512, FoV 146mmx180mm, FA 150°, THK 2mm.<br>T2-FlAIR: TE 108ms, TR 9000ms, TI 2500ms, ETL 16, Matrix size 250x320, FoV 140mmx180mm, FA 150°,THK 2mm.<br>Susceptibility weighted imaging: TE 20ms, TR 27ms,ETL 1, Matrix size 224x256, FoV 157mmx180mm, FA 15°, THK 1.25mm.<br>Multistab 3D TOF MRA imaging: TE 3.69ms, TR 21ms, ETL 1, Matrix size 331x384, FoV 181mmx199mm, FA 18°, THK 0.6mm.<br>RESOLVE-DWI: TE 75ms, TR 10490ms, ETL 0, matrix size 120x160, FoV 156mmx 220mm, FA 180°, THK 2mm.<br>CUSP90-SMS: TE 98ms, TR 4200ms, ETL 59, Matrix size 160x160, FoV 220mmx220mm, FA 90°, THK 2mm.<br>SVS: TE 100ms, TR 9360ms, ETL 18, Matrix size 291x512, FoV 146mmx180mm, FA 150°, THK 2mm.
Area of acquisition	Whole brain

Diffusion MRI ☒ Used ☐ Not used

Parameters	RESOLVE-DWI: TE 75ms, TR 10490ms, ETL 0, matrix size 120x160, FoV 156mmx 220mm, FA 180°, THK 2mm.<br>CUSP90-SMS: TE 98ms, TR 4200ms, ETL 59, Matrix size 160x160, FoV 220mmx220mm, FA 90°, THK 2mm.

Preprocessing

Preprocessing software	Scanner was a Skyra in the Boston Children's Clinical Building. Clinical protocols were used and data reviewed on Synapse 5 Viewer.
Normalization	These pigs were scanned on a clinical scanner using standard clinical protocols. No normalization was performed.
Normalization template	Not applicable
Noise and artifact removal	Scans were visually monitored for motions.
Volume censoring	Not applicable.

Statistical modeling & inference

Model type and settings	Areas of enhancement on axial and coronal T2 and diffusion coefficient images were manually processed on a voxel-per-voxel basis and outlined (itk-SNP software application, Penn Image Computing and Science Laboratory, University of Pennsylvania, Pennsylvania, and Scientific Computing and Imaging Institute, University of Utah, Utah) by the Department of Radiology at Boston Children's Hospital, all of whom were also blinded to treatment allocation. From these values, total volumes of cranial injury were calculated using software normalized to brain volume.
Effect(s) tested	The total volume of injury was compared between groups by t-test.

Specify type of analysis: ☒ Whole brain ☐ ROI-based ☐ Both

Statistic type for inference (See Eklund et al. 2016)	Injury determined based on voxel-per-voxel analysis.
Correction	NA

Models & analysis

n/a	Involved in the study
☒	☐ Functional and/or effective connectivity
☒	☐ Graph analysis
☒	☐ Multivariate modeling or predictive analysis
